# Supplementary material for: A randomized feasibility trial comparing four antimalarial drug regimens to induce Plasmodium falciparum gametocytemia in the controlled human malaria infection model
Source: eLife. 2018 Feb 27;7:e31549. doi: 10.7554/eLife.31549 (PMC5828662; doi:10.7554/eLife.31549)
Supplement: Source code 2. [file elife-31549-code2.docx]

| **Variables and parameters**  d = 1/gametocyte lifespan in density model (logd = natural logarithm of d)  G0 = gametocyte density on day 0 (logG0 = natural logarithm of G0)  loggam_m = natural logarithm of density of male gametocytes  loggam_f = natural logarithm of density of female gametocytes  zero_m = indicator variable for censored observation for male gametocyte density– ie male gametocyte density measured as zero so assumed below detection threshold.  zero_f = indicator variable for censored observation for female gametocyte density– ie female gametocyte density measured as zero so assumed below detection threshold.  varre = variance of subject level random effect (logvarre = its natural logorathm)  varloggamd = variance of log gametocyte density around predicted value (logvarloggamd = its natural logarithm)  sdloggamd = standard deviation of variance of log gametocyte density around predicted value (ie square root of varloggamd)  lg0 = mean predicted log gametocyte density  e = subject level random effect  loggamd = subject specific predicted log gametocyte density  ll = log likelihood  **Model code**  proc nlmixed data=**’datasetname’** alpha=0.05;  parms logd=-2 logvarre=0 logG0=5 logvarloggamd=0 ;  d = exp(logd);  G0=exp(logG0);  varre = exp(logvarre);  varloggamd=exp(logvarloggamd);  sdloggamd = sqrt(varloggamd);  lg0= log(exp(-d*day)*G0);  loggamd = e + lg0;  if (zero_m=0) then ll=-0.5*logvarloggamd-(1/(2*varloggamd))*(loggam_m-loggamd)**2;  else ll=log(probnorm((loggam_m-loggamd)/sdloggamd));  model loggam_m~general(ll);  random e ~normal(0, varre) subject=id;  estimate "mean male gct circulation time" 1/exp(logd);  run;  proc nlmixed data=**’datasetname**’ alpha=0.05;  parms logd=-2 logvarre=0 logG0=5 logvarloggamd=0 ;  d = exp(logd);  G0=exp(logG0);  varre = exp(logvarre);  varloggamd=exp(logvarloggamd);  sdloggamd = sqrt(varloggamd);  lg0= log(exp(-d*day)*G0);  loggamd = e + lg0;  if (zero_f=0) then ll=-0.5*logvarloggamd-(1/(2*varloggamd))*(loggam_f-loggamd)**2;  else ll=log(probnorm((loggam_f-loggamd)/sdloggamd));  model loggam_f~general(ll);  random e ~normal(0, varre) subject=id;  estimate "mean female gct circulation time" 1/exp(logd);  run; |
| --- |

**Supplementary File 7. SAS code used for estimation of gametocyte half-life for gametocytes**
