# Supplementary material for: A randomized feasibility trial comparing four antimalarial drug regimens to induce Plasmodium falciparum gametocytemia in the controlled human malaria infection model
Source: eLife. 2018 Feb 27;7:e31549. doi: 10.7554/eLife.31549 (PMC5828662; doi:10.7554/eLife.31549)
Supplement: Supplementary file 1. [file elife-31549-supp1.docx]

| **Subject** | **Study arm** | **Day of T1** | **Day of T2** | **Parasite density T1** (*Pf*/mL) | **Peak parasite density** (*Pf*/mL) | **Peak gametocyte density** (gct/mL) | **Day of peak gametocytemia** | **Days of asexual parasitemia** | **Duration gametocytemia*** | **AUC asexual** | **AUC sexual** |
| --- | --- | --- | --- | --- | --- | --- | --- | --- | --- | --- | --- |
| Subject ED | LD-SP/SP | 13.3 | 21 | 4442 | 7877 | 368 | 23 | 5 | 24 | 8700 | 3695 |
| Subject DS | LD-SP/SP | 12.3 | 21 | 294 | 20261 | 62 | 23 | 7 | 13 | 16337 | 552 |
| Subject DV | LD-SP/SP | 9.3 | 21 | 1178 | 5057 | 11 | 25 | 3 | 1 | 4280 | 128 |
| Subject DB | LD-SP/SP | 12.8 | 21 | 7877 | 1050 | 14 | 24 | 6 | 2 | 1120 | 86 |
| Subject DO | LD-SP/PIP | 10.8 | 21 | 959 | 2590 | 13 | 19 | 5.5 | 2 | 2773 | 124 |
| Subject EB | LD-SP/PIP | 10.8 | 21 | 5944 | 20134 | 26 | 23 | 5 | 5 | 15225 | 320 |
| Subject DK | LD-SP/PIP | 11.8 | 21 | 4285 | 12617 | 101 | 21 | 6 | 7 | 11334 | 885 |
| Subject EN | LD-SP/PIP | 10.8 | 21 | 24536 | 50210 | 33 | 23 | 7 | 14 | 43777 | 350 |
| Subject DF | LD-PIP/PIP | 10.3 | 14.3 | 681 | 2408 | 70 | 27 | 8 | 25 | 5408 | 715 |
| Subject DX | LD-PIP/PIP | 10.3 | 12.3 | 4155 | 21565 | 96 | 29 | 7.5 | 12 | 24898 | 917 |
| Subject DY | LD-PIP/PIP | 10.3 | 21 | 5781 | 13432 | 46 | 27 | 5.5 | 16 | 12886 | 382 |
| Subject EF | LD-PIP/PIP | 12.3 | 14.3 | 3856 | 9773 | 99 | 27 | 9 | 18 | 15807 | 1348 |
| Subject DL | LD-PIP/SP | 12.3 | 14.3 | 10108 | 63113 | 1285 | 23 | 9 | 25 | 82973 | 9725 |
| Subject EA | LD-PIP/SP | 14.3 | 19.3 | 1266 | 8501 | 755 | 38 | 8 | 25 | 12162 | 10303 |
| Subject DN | LD-PIP/SP | 12.8 | 14.3 | 2070 | 3976 | 199 | 24 | 6.5 | 17 | 4572 | 1582 |
| Subject EK | LD-PIP/SP | 12.8 | 14.3 | 434 | 8480 | 498 | 24 | 7 | 24 | 13332 | 3606 |
|  |  |  |  |  |  |  |  |  |  |  |  |
| *Maximum number of consecutive days of *Pfs25* qRT-PCR measured gametocytemia ≥ 5 gct/mL. | | | | | | | | | | | |

**Supplementary File 1. Individual data of the participants included in analysis.**
