# Supplementary material for: A randomized feasibility trial comparing four antimalarial drug regimens to induce Plasmodium falciparum gametocytemia in the controlled human malaria infection model
Source: eLife. 2018 Feb 27;7:e31549. doi: 10.7554/eLife.31549 (PMC5828662; doi:10.7554/eLife.31549)
Supplement: Supplementary file 2. [file elife-31549-supp2.docx]

| **Gene ID** | **Gene alias** | **Chromosome** | **Forward primer** | **Reverse primer** |
| --- | --- | --- | --- | --- |
| *Pf3D7_1031000* | *Pfs25* | 10 | GAAATCCCGTTTCATACGCTTG | AGTTTTAACAGGATTGCTTGTATCTAA |
| *Pf3D7_1469900* | *PfMGET* | 14 | CGGTCCAAATATAAAATCCTG | GTGTTTTTAATGCTGGAGCTG |

**Supplementary File 2. Selected *P. falciparum* gene targets and primers of qRT PCR assays**
