# Supplementary material for: A randomized feasibility trial comparing four antimalarial drug regimens to induce Plasmodium falciparum gametocytemia in the controlled human malaria infection model
Source: eLife. 2018 Feb 27;7:e31549. doi: 10.7554/eLife.31549 (PMC5828662; doi:10.7554/eLife.31549)
Supplement: Supplementary file 3. — The table shows for each target: limit of detection (LOD; defined as lowest pathogen concentration with reproducible detection); limit of quantification (LOQ; defined as lowest pathogen concentration where the CV was <5%), slope, efficiency (E), and the coefficient of correlation of combined trendlines (R2). [file elife-31549-supp3.docx]

| **Target** | **LOD parasites/mL** | **LOQ parasites/mL** | **Slope** | **E (%)** | **R^2^** |
| --- | --- | --- | --- | --- | --- |
| *Pfs25* | 5 | 5 | -3.35 | 98.7 | 0.99 |
| *PfMGET* | 20 | 20 | -3.51 | 92.9 | 0.99 |
| *18S* | 20 | 20 | -3.47 | 94.1 | 0.99 |

**Supplementary File 3. Quality parameters of qRT PCR and qPCR**

The table shows for each target: limit of detection (LOD; defined as lowest pathogen concentration with reproducible detection); limit of quantification (LOQ; defined as lowest pathogen concentration where the CV was <5%), slope, efficiency (E), and the coefficient of correlation of combined trendlines (R^2^).
