# Supplementary material for: A randomized feasibility trial comparing four antimalarial drug regimens to induce Plasmodium falciparum gametocytemia in the controlled human malaria infection model
Source: eLife. 2018 Feb 27;7:e31549. doi: 10.7554/eLife.31549 (PMC5828662; doi:10.7554/eLife.31549)
Supplement: Supplementary file 4. [file elife-31549-supp4.pdf]

CONFIDENTIAL

CLINICAL TRIAL PROTOCOL

(15 April 2016)

Controlled Human Malaria Infection model for evaluation of transmission-blocking interventions – Study 1

Version 3.0, 15-April-2016

(“*CHMI-trans1*”)

PROTOCOL TITLE: **'**Controlled Human Malaria Infection study to assess gametocytaemia and mosquito transmissibility in participants challenged with *Plasmodium falciparum* by sporozoite challenge to establish a model for the evaluation of transmission-blocking interventions'

**Protocol ID**	**CHMI-trans1**
**Short title**	Controlled Human Malaria Infection model for evaluation of transmission-blocking interventions – Study 1
**Toetsingonlinenummer**	NL56659.091.16
**EudraCT number**	*2016-001379-66*
**Version**	**3.0**
**Date**	**15-04-2016**
**Project leader/Clinical investigator**	**Isaie J. Reuling, M.D.**<br>Radboudumc, Department of Medical Microbiology<br>Tel: +31 (0)24 3619515<br>Fax: +31 (0)24 3614666<br>E-mail: isaie.reuling@radboudumc.nl
**Sponsor**<br><br><br><br><br><br>**Principal Investigator**<br><br><br><br><br><br>**Collaborating principal investigator**	Stichting Katholieke Universiteit<br>Roadboud University Medical Centre (Radboudumc)<br>Department of Medical Microbiology<br>Geert Grooteplein-Zuid 10<br>6525 GA, Nijmegen, The Netherlands<br><br>**Prof. Robert W. Sauerwein, M.D.**<br>Radboudumc, Department of Medical Microbiology<br>Tel: +31243610577<br>Fax: +31243614666<br>e-mail: robert.sauerwein@radboudumc.nl<br><br>**Dr. Teun Bousema**<br>Radboudumc, Department of Medical Microbiology<br>Tel: +31243617574<br>Fax: +31243540216<br>e-mail: teun.bousema@radboudumc.nl

**Funding partner**	**PATH**<br><br>2201 Westlake Avenue, Suite 200<br>Seattle, Washington<br>98121, USA
**Clinical supervisors**	**Prof. André van der Ven, M.D.**<br>Radboudumc, Department of Internal Medicine<br>Tel: +31243686791<br>e-mail: andre.vanderven@radboudumc.nl<br><br>**Dr. Quirijn de Mast, M.D.**<br>Radboudumc, Department of Internal Medicine<br>Tel: +31243610929<br>e-mail: quirijn.demast@radboudumc.nl
**Biological evaluator**	**Dr. Rob Hermsen, PhD**<br>Radboudumc, Department of Medical Microbiology<br>Tel: +31 (0)24 3613663<br>E-mail: Rob.Hermsen@radboudumc.nl<br><br>**Dr. Anja Scholzen, PhD**<br>Tel: +31 (0)24 3614665<br>E-mail: Anja.Scholzen@radboudumc.nl
**Independent expert (s)**	Radboudumc<br>Department of Internal Medicine<br>**Dr. Monique Keuter**<br>Tel : +31 (0)24 3614462<br>Monique.Keuter@radboudumc.nl
**Laboratory sites**	**Radboudumc Clinical Chemical Laboratory**<br>**Jan Verhagen**<br>Tel: +31 (0)24 3616997<br>Fax: +31 (0)24 3568408<br>e-mail: j.verhagen@akc.umcn.nl<br><br>**Clinical Microbiology Laboratory**<br>**Prof.dr. Heiman Wertheim**<br>Tel: +31 (0)24 36 14281<br>e-mail: Heiman.Wertheim@radboudumc.nl

**Pharmacy**	**Radboudumc Clinical Pharmacy**<br>Clinical Trial Unit<br>**Dr. Rob ter Heine**<br>Tel: +31 (0)24 3616405<br>Fax: +31 (0)24 3668755<br>e-mail: R.terHeine@radboudumc.nl
**External monitor**	**CROMSOURCE**<br>Lange Dreef 11H<br>4131 NJ Vianen, The Netherlands<br>Tel: +31 347 344977 ext. 2614<br>Fax: +31 347 34 42 89<br>Mobile: +31 6 13516468

PROTOCOL SIGNATURE SHEET

Investigator Agreement

"I have read this protocol and agree to abide by all provisions set forth therein.

I agree to comply with the principles of the International Conference on Harmonisation Tripartite Guideline on Good Clinical Practice."

Name	Signature	Date
Sponsor and principal investigator:<br>***Robert Sauerwein, MD, PhD***<br>*Department of Medical Microbiology*<br>*Radboud university medical center*	[signature]	15/4/16
Collaborating principal investigator:<br>***Teun Bousema, PhD***<br>*Department of Medical Microbiology*<br>*Radboud university medical center*	[signature]	15/4/16
Project leader:<br>***Isaie Reuling, MD***<br>*Department of Medical Microbiology*<br>*Radboud university medical center*	[signature]	15/4/16

Version History

Version	Date	Author(s)	Summary of changes
2.0	15 March 2016	Isaie Reuling<br>Teun Bousema<br>Robert Sauerwein	- Piperaquine and sulfadoxine-pyrimethamine are investigational products (section 6).<br>- Investigator's Brochure of piperaquine added as reference (attachment D2g).<br>- Added detailed drug and dose selection rationale (section 6.4).<br>- Added information of potential risks of subcurative treatment (section 6.5).<br>- Added information on SUSARs (section 9.2.3).<br>- Added information on drug interactions (section 13.1.1.h).
3.0	15 April 2016	Isaie Reuling<br>Teun Bousema<br>Robert Sauerwein	- Minor revision in section 4.4 Sample size calculation.

TABLE OF CONTENTS

1. INTRODUCTION AND RATIONALE 16
   1.1 Introduction 16
   1.2 Rationale 17
   1.3 Clinical Experience 18
   1.4 Safety 19
2. OBJECTIVES 20
3. STUDY DESIGN 21
4. STUDY POPULATION 23
   4.1 Population (base) 23
   4.2 Inclusion criteria 23
   4.3 Exclusion criteria 24
   4.4 Sample size calculation 25
5. TREATMENT OF SUBJECTS 27
   5.1 Investigational product/treatments (for DT1 and DT2) 27
   5.2 Use of co-intervention 27
   5.3 Escape medication (if applicable) 27
6. INVESTIGATIONAL PRODUCT (TREATMENT PRODUCT) 28
   6.1 Name and description of investigational products 28
   6.2 Summary of findings from non-clinical studies 28
   6.3 Summary of findings from clinical studies 28
      6.3.1 Clinical data for sulfadoxine-pyrimethamine 28
      6.3.2 Clinical data for Piperaquine 29
      6.3.3 Pharmacokinetics and Metabolism of sulfadoxine-pyrimethamine 29
      6.3.4 Pharmacokinetics and Metabolism of Piperaquine 29
      6.3.5 Safety and tolerability of sulfadoxine-pyrimethamine 30
      6.3.6 Safety and tolerability of Piperaquine 30
   6.4 Drug and dose selection rationale 31
   6.5 Potential Risks 32
   6.6 Potential Benefits 33
   6.7 Risk Management 33
   6.8 Preparation and labeling of Investigational Medicinal Product 33
   6.9 Drug accountability 34
NON-INVESTIGATIONAL PRODUCT (CHALLENGE PRODUCT) 35
   6.10 Name and description of non-investigational product(s) 35
   6.11 Summary of findings from non-clinical studies 35
   6.12 Summary of findings from clinical studies 35
   6.13 Summary of known and potential risks and benefits 36
   6.14 Description and justification of route of administration and dosage 36
   6.15 Dosages, dosage modifications and method of administration 37
   6.16 Preparation and labeling of Non Investigational Medicinal Product 37
   6.17 Drug accountability 37
7. METHODS 38

7.1 Study parameters/endpoints 38
7.1.1 Main study parameter/endpoint 38
7.1.2 Secondary study parameters/endpoints 38
7.1.3 Exploratory study parameters/endpoints 38
7.2 Randomisation, blinding and treatment allocation 38
7.3 Study procedures 39
7.3.1 Screening period: Screening, Inclusion and Baseline visits 39
7.3.2 Controlled Human Malaria Infection 39
7.3.3 Treatment with sulfadoxine-pyrimethamine (DT1 and DT2) 42
7.3.4 Treatment with piperaquine (DT1 and DT2) 42
7.3.5 Treatment with Malarone® (ET) 42
7.3.6 Physical examination 43
7.3.7 Vital signs 43
7.3.8 Patient-reported outcomes (study diary) 43
7.3.9 Electrocardiogram 44
7.3.10 Blood sampling and safety laboratory evaluations 44
7.3.11 Analysis of asexual parasite densities after challenge infection 45
7.3.12 Direct Membrane Feeding Assays (DMFA) 45
7.3.13 Quantification of gametocytes, gametocyte sex ratio and gametocyte infectivity. 46
7.3.14 Immunological assays 47
7.3.15 Case report forms and data collection 47
7.3.16 Flowchart Study Design 48
7.4 Withdrawal of individual subjects 50
7.5 Replacement of individual subjects after withdrawal 51
7.6 Follow-up of subjects withdrawn from treatment 51
7.7 Premature termination of the study 51
8. SAFETY REPORTING 52
8.1 Temporary halt for reasons of subject safety 52
8.2 AEs, SAEs and SUSARs 52
8.2.1 Adverse events (AEs) 52
8.2.2 Serious adverse events (SAEs) 52
8.2.3 Suspected unexpected serious adverse reactions (SUSARs) 53
8.3 Follow-up of (serious) adverse events 54
8.3.1 Adverse event data collection 54
8.3.2 Assessment of causality 54
8.3.3 Follow-up of adverse events 55
8.4 Local Safety Monitor (LSM) and Safety Monitoring Committee (SMC) 55
8.4.1 Local safety monitor 55
8.4.2 Safety Monitoring Committee (SMC) 55
8.4.3 Review of safety data by the safety monitor and SMC 56
8.4.4 Safety stopping rules 56
9. STATISTICAL ANALYSIS 57

9.1 Primary study parameter(s) 57
9.2 Secondary study parameter(s)....................58
9.3 Other study parameters 58
10. ETHICAL CONSIDERATIONS 59
10.1 Regulation statement 59
10.2 Recruitment and consent 59
10.3 Benefits and risks assessment, group relatedness 60
10.3.1 Ethical aspects concerning the production of *P. falciparum* infected mosquitoes 60
10.3.2 Ethical aspects concerning the use of human volunteers....................60
10.4 Compensation for injury 61
10.5 Incentives....................61
11. ADMINISTRATIVE ASPECTS, MONITORING AND PUBLICATION....................63
11.1 Handling and storage of data and documents 63
11.1.1 Confidentiality 63
11.1.2 Data collection 63
11.1.3 Database management and quality control 63
11.2 Monitoring and Quality Assurance 64
11.3 Amendments....................64
11.4 Annual progress report 64
11.5 Temporary halt and (prematurely) end of study report 65
11.6 Public disclosure and publication policy....................65
12. STRUCTURED RISK ANALYSIS 66
12.1 Controlled Human Malaria Infection....................66
12.1.1 Potential issues of concern 66
12.1.2 Cardiac events following Controlled Human Malaria Infections....................68
12.1.3 Synthesis 71
13. REFERENCES 72

LIST OF ABBREVIATIONS AND RELEVANT DEFINITIONS

A.	*Anopheles*
ACT	Artemisinin-Based Combination Treatment
AE	Adverse Event
AL	artemether–lumefantrine
ALT	Alanine Aminotransferase
ANOVA	analysis of variance
AR	Adverse Reaction
BMI	Body mass index
BP	Blood pressure
BSL	Biosafety Level
CA	Competent Authority
CCMO	Central Committee on Research Involving Human Subjects; in Dutch: Centrale Commissie Mensgebonden Onderzoek
CHMI	Controlled Human Malaria Infection
CHMI-trans	Controlled Human Malaria Infection Transmission Model
CRF	Case Report Form
CRO	Contract Research Organization
CV	Curriculum Vitae
DSF	Direct Skin Feeding
DHA	dihydroartemisinine
DHA-PQP	Dihydroartemisinine - piperaquine phosphate
DMFA	Direct Membrane Feeding Assay
DT1	Day of Treatment 1 (subcurative treatment)
DT2	Day of Treatment 2 (curative treatment)
ECG	ElektroCardioGram
EDTA	Ethylenediaminetetraacetic acid
ET	End Treatment with Malarone®
eCRF	electronic Case Report Form
ELISA	Enzyme-Linked Immuno Sorbent Assay
G6PD deficiency	Glucose-6-phophatedehydrogenase deficiency
GCP	Good Clinical Practice
GP	General Practitioner
HBV	Hepatitis B Virus
HCV	Hepatitis C Virus

**HDPE**	**high density polyethylene**
**HIV**	**Human Immunodeficiency Virus**
**HTLV**	**HumanT-lymphotropic Virus**
**IC**	**Informed Consent**
**IFN-γ**	**Interferon-gamma**
**IL-1β**	**Interleukin 1β**
**IL-6**	**Interleukin 6**
**IPTp**	**intermittent preventive treatment of malaria in pregnancy**
**IRB**	**Institutional Review Board**
**ITN**	**insecticide-impregnated bednets**
**IV**	**Intravenous**
**LDH**	**Lactate dehydrogenase**
**LSM**	**Local Safety Monitor**
**mAbs**	**Monoclonal antibodies**
**METC**	**Medical research ethics committee (MREC); in Dutch: medisch ethische toetsing commissie (METC)**
**MFS**	**Membrane Feed for Sporozoite production**
**NaCl**	**Sodium Chloride**
**NF54**	**Nijmegen *falciparum* strain 54**
**NK cells**	**Natural killer cells**
P.	***Plasmodium***
**par/ml or p/ml**	**parasites per milliliter**
**PATH REC**	**PATH Research Ethics Committee; the funding partner's ethical committee.**
**PBMC**	**Peripheral blood mononuclear cell**
**PCR**	**Polymerase Chain Reaction**
**Pf**	***Plasmodium falciparum***
**PhHV**	**Phocine Herpes Virus**
**Pip**	**piperaquine**
**qPCR**	**Real-time Quantitative Polymerase Chain Reaction**
**qRT-PCR**	**Quantitative Reverse Transcriptase Polymerase Chain Reaction**
**QT-NASBA**	**Quantitative Nucleic Acid Sequence Based Amplification**
**Radboudumc**	**Radboud university medical center**
**SAE**	**Serious Adverse Event**
**Sanquin**	**Sanquin Blood Supply Foundation, who on the basis of the Blood Supply Act is responsible for all blood supply (blood and blood**

	**products) for transfusion in The Netherlands.**
**SCORE**	**Systematic Coronary Risk Evaluation**
**SMC**	**Safety Monitoring Committee**
**SMFA**	**Standard Membrane Feeding Assay**
**SOP**	**Standard Operating Procedure**
**SP**	**sulfadoxine-pyrimethamine**
**SPC**	**Summary of Product Characteristics**
**Sponsor**	**The sponsor is the party that commissions the organisation or performance of the research, for example a pharmaceutical company, academic hospital, scientific organisation or investigator. A party that provides funding for a study but does not commission it is not regarded as the sponsor, and is referred to here as a funding partner.**
**SWAB**	**Stichting Werkgroep Antibioticabeleid**
**SUSAR**	**Suspected Unexpected Serious Adverse Reaction**
**TBV(s)**	**Transmission Blocking Vaccine(s)**
**TNF-α**	**Tumor Necrosis Factor alpha**
**WIRB**	**Western Institutional Review Board; PATH's designated IRB to who PATH REC delegates ethical review and oversight of clinical studies.**
**WHO**	**World Health Organization**
**WMO**	**Medical Research Involving Human Subjects Act (in Dutch: Wet Medisch-wetenschappelijk Onderzoek met Mensen**
**ZAVIN**	**Ziekenhuis Afval Verwerkings Installatie Nederland (Hospital Waste Disposal company)**
**γGT**	**Gamma Glutamyl Transferase**

SUMMARY

Rationale:

Malaria is one of the most devastating infectious diseases worldwide. Despite all the progress that has been made in reducing the malaria burden, in 2013 there were still ~200 million cases and ~0.6 million deaths, mainly in children less than five years of age[1]. In addition to the intolerable clinical burden, malaria forms a profound economic burden for the affected countries, which are already struggling with poverty. The urgency of the situation is further emphasized by the waning effectiveness of all currently registered anti-malarials due to fast emergence and spread of resistance and the absence of an highly effective vaccine[2].

Malaria transmission blocking vaccines (TBVs) and transmission-blocking drugs aim to interrupt the development of parasites in the mosquito[3]. TBVs will play a central role in efforts to reduce the malaria burden, to contain drug resistance and to move towards malaria elimination[2, 4].

The clinical development of such transmission blocking interventions will be greatly accelerated by a suitable model for their evaluation.

Controlled Human Malaria Infections (CHMI) are an established model for evaluation of malaria candidate vaccines and drugs targeting pre-erythrocytic or asexual blood stages.

The primary aim of this project is to develop a controlled human malaria infection transmission model ("*CHMI-trans*") or "challenge model" to evaluate the capacity of vaccines, biologics (monoclonal antibodies, or mAbs), and drugs to block malaria parasite transmission by assessing infectiousness of *Plasmodium falciparum* (*Pf*) gametocyte carriers for *Anopheles* mosquitoes.

Objective:

Primary objectives:

1) To evaluate the safety of four different CHMI-trans protocols in healthy malaria-naïve volunteers challenged with *Plasmodium falciparum* by sporozoite challenge.
2) To determine the best CHMI-trans protocol for induction of stable gametocytaemia at densities detectable by qRT-PCR

Secondary objectives:

3) To determine the dynamics of gametocyte commitment, maturation and sex ratio by molecular markers of sexual stage development.
4) To determine the time-point of peak density of gametocytaemia in the CHMI-trans model.

Exploratory Objectives:

5) To assess gametocyte infectiousness for *Anopheles* mosquitoes through mosquito feeding assay (Direct Membrane Feeding Assay, DMFA).
6) To determine gametocyte fitness by molecular markers, and *ex vivo* assessments of gamete formation and fertilization.

Study design:

Single center, open label, randomized, sporozoite challenge study.

Study population:

A maximum of 32 healthy volunteers, aged 18 to 35 years, male and female, will participate in the study.

Intervention:

A total of 32 volunteers will be randomly assigned to four groups (n=8) and subjected to a standard controlled human malaria infection (CHMI) delivered by five *Pf*-infected mosquitoes (3D7 clone) (see Figure 1, section 3). Treatment is subsequently initiated to induce gametocytaemia (treatment 1, DT1) and to clear pathogenic asexual parasites whilst leaving gametocytes unaffected (treatment 2, DT2). At the end of the study, treatment of all parasite stages is provided following national treatment guidelines (end treatment, ET).

Once malaria infections are detected by 18S qPCR positive at a density of 5,000 par/ml (day of treatment 1 [DT1]), groups 1 and 2 will be treated with a course of subcurative sulfadoxine-pyrimethamine (SP) (SP low, 500mg/25mg). Groups 3 and 4 will receive piperaquine (Pip) in a low-dose (Pip low, 480 mg). Daily blood samples will allow detailed quantification of gametocytes, gametocyte sex ratio and *ex vivo* assessments of gametocyte fitness. Using blood samples taken twice daily, the initial clearance of parasitaemia will be carefully monitored. After DT1, volunteers will receive a curative treatment (DT2) when a recrudescence of asexual parasitaemia occurs or on day 21 post challenge infection, whichever comes first. Recrudescence of asexual parasitaemia will be carefully monitored until parasite densities reach 1,500 par/ml by 18S qPCR, at which time participants will receive the curative treatment (DT2). Volunteers in **group 1 (SP low/SP high)** will be treated with sulfadoxine-pyrimethamine (1000mg/50mg) and **group 2 (SP low/Pip high)** with piperaquine (960mg). Volunteers in **group 3 (Pip low/Pip high)** will be treated with piperaquine (960mg) and **group 4 (Pip low/SP high)** with sulfadoxine-pyrimethamine (1000mg/50mg). These treatment regimens for DT2 have been shown to cure asexual parasitaemia while leaving immature and mature gametocytes unaffected[5]. To ensure the radical clearance of all parasite stages, all volunteers will receive a final treatment (ET)

according to national guidelines with atovaquone/proguanil (Malarone®) on day 42. In case a volunteer remains 18S qPCR and Pfs25 qRT-PCR negative for 7 days after DT1, final treatment with Malarone® will also be initiated and end of study will apply for the volunteer. On the day of administration of DT2 and four days after initiation of DT2, venous blood samples will be obtained for Direct Membrane Feeding Assay (DMFA). An additional blood sample will be taken for DMFA between day 7-21 after DT2, the exact time-point being based on the density of gametocytaemia as measured by Pfs25 qRT-PCR. These blood samples will be offered to 300 mosquitoes to provide preliminary evidence on the infectivity of volunteers at these time-points.

Main study parameters/endpoints:

Primary endpoints:

- Frequency and magnitude of adverse events in the CHMI-trans model in study groups.
- Prevalence of gametocytes in the CHMI-trans model in study groups.

Secondary endpoints:

- Peak density and time-point of peak density of gametocytes by qRT-PCR.
- The area under the curve of gametocyte density versus time.
- Assessment of the dynamics of gametocyte commitment, maturation and sex-ratio.

Exploratory endpoints

- Gametocyte infectivity to *Anopheles* mosquitoes by DMFA.
- Gamete formation and fertilization as ex vivo indicators of gametocyte fitness

Nature and extent of the burden and risks associated with participation, benefit and group relatedness:

Benefits: There are no direct benefits for volunteers. Risks: Risks for volunteers are related to (i) exposure to *P. falciparum* malaria infection, (ii) potential side-effects associated with treatment medications.

Burden:

The study represents a challenge infection by bites of 5 (3D7 *P. falciparum*) infected mosquitoes. After the challenge there will be a period (42 days) of intense clinical monitoring with frequent site visits (up to two times a day) and blood examinations. Depending on the group, the subjects will receive a sub-curative treatment (DT1) with either sulfadoxine-pyrimethamine or piperaquine when 18S qPCR positive at 5000 par/ml (threshold of microscopic detection). Using blood samples taken twice daily, the initial clearance of parasitaemia will be carefully monitored. After DT1, volunteers will receive a curative treatment (DT2) when a recrudescence of asexual parasitaemia occurs or on day 21 post challenge infection, whichever comes first. Recrudescence of asexual parasitaemia will be carefully monitored until parasite densities reach 1,500 par/ml by 18S qPCR, at which time participants will receive a curative dose of sulfadoxine-pyrimethamine or piperaquine (DT2) depending on the study group to provide clearance of asexual parasites. All volunteers will receive a final treatment (ET) according to national guidelines with Malarone® on day 42 to assure the radical clearance of all parasite stages. In case a volunteer remains 18S qPCR and Pfs25 qRT-PCR negative for 7 days after DT1, final treatment with Malarone® will also be initiated and end of study will apply for the volunteer. The exact number of site visits and blood examinations per volunteers depends on the time to positive 18S qPCR above 5000 parasites/ml and potential recrudescence - with a maximum number of 50 study visits and a maximum of 500 mL collected blood. In addition periodical physical examinations will be performed and the subject is asked to complete a diary. The duration of subject participation will be 64 days from day of challenge, following a screening period of up to 120 days.

1. INTRODUCTION AND RATIONALE

1.1 Introduction

Malaria is one of the most devastating infectious diseases worldwide. Despite all the progress that has been made in reducing the burden of malaria by the up-scaling of protective measures and efficacious treatment[6], in 2013 there were still ~200 million cases and ~0.6 million deaths, particularly of children less than five years of age[1]. In addition to the appalling human suffering, this disease forms a profound economic burden for the affected countries, which are already struggling with poverty. The urgency of the situation is further emphasized by the waning effectiveness of all currently registered anti-malarials due to fast emergence and spread of resistance and the absence of an effective vaccine[2, 7].
The World Health Organization (WHO) has declared malaria control a global development priority and has changed their recommendation from control programs to eradication programs. It is widely accepted that malaria eradication is unlikely to be attainable with the currently available tools[6, 8].

The major challenge for malaria elimination is the highly efficient spread of malaria parasites. Human malaria is caused by protozoa of the genus *Plasmodium*: *P. falciparum*, *P. vivax*, P. *ovale*, *P. malariae*, and *P. knowlesi*. Parasites (sporozoite stage) are injected into the skin by an infected female *Anopheles* mosquito. After penetration of the skin capillaries, sporozoites are transported to the liver, where they develop and multiply in liver cells before release into the blood (merozoite stage) and invading red blood cells for further maturation and multiplication. The cyclical proliferation of asexual stages within the human red blood cells is responsible for the occurrence of clinical symptoms. A small fraction of asexual stages is committed to enter sexual development. The formation of male and female gametocytes are essential for parasite transmission via the female *Anopheles* mosquito vector. Circulating gametocytes do not cause clinical pathology or symptoms. The circulating gametocytes therefore have no clinical consequences but play an essential role in the onward transmission of malaria infections. The renewed focus on malaria elimination has increased the priority of research towards development of interventions to block malaria transmission, including transmission blocking vaccines (TBVs). By interrupting transmission of malaria parasites in mosquito vectors, a reduction in the number of secondary infections in the community is expected. TBVs will play an important role in complete arrest of malaria transmission in endemic areas[4, 9]. Similarly, a number of gametocidal and/or sporontocidal drug candidates have been generated in recent years[10]. From a community perspective, deployment of transmission-blocking drugs and TBVs will be an efficient complementary

element in an integrated program of anti-malarial interventions, particularly for malaria elimination.

1.2 Rationale

Controlled human malaria infection (CHMI) studies have become a safe[11-13] and widely accepted model for evaluating the efficacy of vaccines[14], anti-malarial drug candidates[15-17], diagnostic assays[18] and assessment of immunologic responses[19, 20]. These studies provide a cost-effective and fast way to circumvent the use of large-scale field efficacy studies for down selection of intervention candidates. However, an effective model to evaluate interventions to block malaria transmission is currently lacking. In this project we aim to develop a controlled human malaria infection transmission model ("*CHMI-trans*") or "challenge model" to, eventually, evaluate the capacity of vaccines, biologics (monoclonal antibodies, or mAbs), and drugs to block malaria parasite transmission by assessing infectiousness of *Plasmodium falciparum* (*Pf*) gametocyte carriers for *Anopheles* mosquitoes.

The first step of malaria parasite transmission from humans to *Anopheles* mosquitoes, is the generation of mature gametocytes in the human peripheral blood. Gametocytes are non-pathogenic malaria life stages and typically comprising less than 5 percent of the total parasite population prior to treatment. Once treatment is initiated, gametocyte production ceases abruptly (e.g. artemisinins) or is tolerated or even stimulated as part of a terminal investment of malaria parasites under drug pressure (e.g. sulfadoxine-pyrimethamine and piperaquine)[5, 21, 22]. Importantly, malaria transmission is not prevented by currently used antimalarial drugs or the recently proposed malaria vaccine RTS,S. Malaria TBVs and drugs can interrupt parasite development in the mosquito and thereby play a central role in malaria elimination efforts and in efforts to contain the spread of drug resistant malaria[4, 23]. However, for downstream selection and clinical development, there is currently an uncertain association between the methods used for preclinical and early clinical evaluation of candidate drugs/vaccines (e.g., the standard membrane feeding assay, or SMFA) and their ultimate field deployment, where the infectivity of naturally exposed hosts forms a key outcome measure. The standard membrane feeding assay (SMFA) can determine the percentage of the transmission-reducing activity (TRA) of human serum by feeding Anopheles mosquitoes on human blood with cultured gametocytes, mixed with experimental sera. Presently, we are heavily reliant on the SMFA to inform the early clinical efficacy stage-gate; however, this assay has not been accurately calibrated against the most widely used ex vivo assessment of gametocyte infectiousness, the direct membrane feeding assay (DMFA)[24]. Therefore, its predictive value in assessing interventions that block human-to-mosquito transmission remains uncertain[3].

In order to use the CHMI model for the evaluation of transmission blocking interventions, current CHMI challenge methods that abort asexual blood-stage parasites prior to emergence of sexual-stage gametocytes need to be modified to optimize gametocyte development and thus transmission to *Anopheles* mosquitoes. Importantly, preliminary data show the presence of gametocytes in the CHMI volunteers after piperaquine treatment, with undetermined infectivity for mosquitoes[25].

Establishment of the *CHMI-trans* model will facilitate effective bridging of SMFA to the direct membrane feeding assay (DMFA) and direct skin feeding assay (DSF) (such as via the evaluation of mAbs with defined transmission-blocking activity), to support the future positioning of SMFA as a more informative tool for predicting clinical outcomes. Most importantly, successful completion of the proposed work will fill a critical unmet need by making available a more biologically relevant assay to rapidly and cost effectively assess transmission-blocking interventions during early clinical development.

1.3 Clinical Experience

There is a large clinical experience with infecting humans by the bite of *P. falciparum* sporozoite-infected mosquitoes. These challenge trials have become highly standardized[26]. The first human malaria challenge study was performed in 1917, and since 1986, when the modern protocol using laboratory adapted *P. falciparum* strains was first performed by the US army, >3,500 subjects have been challenged by the bites of mosquitoes fed on cultures of *P. falciparum* gametocytes to produce sporozoites[27]. Infecting humans by the bite of *P. falciparum* sporozoite-infected mosquitoes is an established clinical trial methodology and is considered a reproducible, predictable and safe method of inducing *P. falciparum* malaria. The results of such studies were summarized in 1997 [11], in 2007 [28] and in 2012 [29].

The Radboud university medical center (Radboudumc) has the experience and infrastructure to conduct CHMI trials. Over 350 subjects have undergone CHMI in studies conducted by Radboudumc since 2001. Standard operating procedures according to international standards are in place for both clinical and laboratory activities, and the Radboudumc mosquito and parasite culture was positively audited in 2014 by an international independent auditor. Radboudumc has developed a sensitive method of parasite detection by real-time quantitative PCR (18S qPCR) in whole blood that allows us to detect malaria infection in an early stage and is able to detect small differences in parasite density[30]. We further pioneered the molecular detection of low densities of gametocytes[31] and sexing of gametocytes[32] assays as parameters for the transmission from humans to mosquitoes[33].

1.4 Safety

After CHMI most malaria-naive volunteers experience symptoms such as headache, chills or fatigue during 1-3 days. During the extensive experience with CHMI, severe or life-threatening malaria has never been reported.

In February 2008 a cardiac serious adverse event (SAE) in a twenty-year-old female participating in an LSA3/Alhydrogel (LSA-3 CMO-07/37; NL14715.000.06) malaria vaccine trial was reported to the CCMO (CCMO08.1096/MA/14715). The findings have been published as a case report titled “Cardiac complication after experimental human malaria infection: case report”, A.E. Nieman et al, Malar. J. 2009 Dec 3;8(1):277.The differential diagnosis was acute coronary syndrome or myocarditis but the true nature and pathophysiology of the event remained unclear.

In February 2013, in the TIP5 study (NL39541.091.12), a 23 year old healthy male volunteer experienced a cardiac SAE. The findings have been published as a case report titled “Ideopathic acute myocarditis during treatment for controlled human malaria infection: a case report”, M.P.A. van Meer et al, Malar. J. 2014, 13: 38. This SAE was diagnosed as myocarditis, while the relation with the malaria infection was not resolved.

In November 2014 a 23 year old healthy male taking part in the BMGF1 study (NL48301.91.14) experienced a cardiac SAE 10 days after a malaria infection under chloroquine prophylaxis (ChemoProphylaxis and Sporozoite immunization). This SAE concerned an asymptomatic hs-troponin T elevation (maximally 168ng/l) diagnosed as a mild myocarditis as reported to the CCMO (AE001.14.48301). Though certain cardiovascular risk factors were present (smoking, recent cannabis use), the temporal relationship with the malaria infection and the previous cases of suspected or confirmed myocarditis after malaria infection, make an association with CHMI likely.

As a result of these cardiac SAEs our safety procedures for CHMI have been strongly intensified. In the current trial, we will adhere to those stringent procedures that are relevant, including:

1. Individuals are excluded from participation if they have first or second degree relatives who had cardiac events under the age of 50
2. A positive urine toxicology test for amphetamines, cocaine and cannabis is an exclusion criterion
3. Increased control of hs troponin T as a marker of cardiac damage; treatment with Malarone® is initiated in consultation with the cardiologist.
4. Daily measurements of platelets; volunteers will be treated with DT1 when platelet levels are <120x 10^9/L and DT2 when platelet levels are <50x 10^9/L.

2. OBJECTIVES

Primary objectives:

1) To evaluate the safety of four different CHMI-trans protocols in healthy malaria-naïve volunteers challenged with *Plasmodium falciparum* by sporozoite challenge.
2) To determine the best CHMI-trans protocol for induction of stable gametocytaemia at densities detectable by qRT-PCR

Secondary objectives:

3) To determine the dynamics of gametocyte commitment, maturation and sex ratio by molecular markers of sexual stage development.
4) To determine the peak density and time-point of peak density of gametocytaemia in the CHMI-trans model.
5) To determine the area under the curve of gametocyte density versus time.

Exploratory Objectives:

6) To assess gametocyte infectiousness for Anopheles mosquitoes through mosquito feeding assay (Direct Membrane Feeding Assay, DMFA).
7) To determine gametocyte fitness by molecular markers, and *ex vivo* assessments of gamete formation and fertilization.

3. STUDY DESIGN

This study will be a single center, open label clinical trial. A total of 32 volunteers will be randomly assigned to four groups (n=8) and subjected to a standard controlled human malaria infection (CHMI) delivered by five *Pf*-infected mosquitoes (3D7 clone) (see Figure 1, section 3). This includes close follow-up after exposure with regular visits to the clinical trial centre, periodic physical examinations, frequent blood sampling and adverse events will be recorded in a diary. For all subjects, during this period all relevant investigations will be carried out on an outpatient basis, including frequent safety analyses.

Treatment is subsequently initiated to induce gametocytaemia (treatment 1, DT1) and to clear pathogenic asexual parasites whilst leaving gametocytes unaffected (treatment 2, DT2). At the end of the study, treatment of all parasite stages is provided following national treatment guidelines (end treatment, ET).

Once malaria infections are detected by 18S qPCR positive at a density of 5,000 par/ml (day of treatment 1 [DT1]), groups 1 and 2 will be treated with a course of subcurative sulfadoxine-pyrimethamine (SP) (SP low, 500mg/25mg). Groups 3 and 4 will receive piperaquine (Pip) in a low-dose (Pip low, 480 mg). Daily blood samples will allow detailed quantification of gametocytes, gametocyte sex ratio and *ex vivo* assessments of gametocyte fitness. Using blood samples taken twice daily, the initial clearance of parasitaemia will be carefully monitored. After DT1, volunteers will receive a curative treatment (DT2) when a recrudescence of asexual parasitaemia occurs or on day 21 post challenge infection, whichever comes first. Recrudescence of asexual parasitaemia will be carefully monitored until parasite densities reach 1,500 par/ml by 18S qPCR, at which time participants will receive the curative treatment (DT2). Volunteers in **group 1 (SP low/SP high)** will be treated with sulfadoxine-pyrimethamine (1000mg/50mg) and **group 2 (SP low/Pip high)** with piperaquine (960mg). Volunteers in **group 3 (Pip low/Pip high)** will be treated with piperaquine (960mg) and **group 4 (Pip low/SP high)** with sulfadoxine-pyrimethamine (1000mg/50mg). These treatment regimens for DT2 have been shown to cure asexual parasitaemia while leaving immature and mature gametocytes unaffected[5]. To ensure the radical clearance of all parasite stages, all volunteers will receive a final treatment (ET) according to national guidelines with atovaquone/proguanil (Malarone®) on day 42. In case a volunteer remains 18S qPCR and Pfs25 qRT-PCR negative for 7 days after DT1, final treatment with Malarone® will also be initiated and end of study will apply for the volunteer. On the day of administration of DT2 and four days after initiation of DT2, venous blood samples will be obtained for Direct Membrane Feeding Assay (DMFA). An additional blood sample will be taken for DMFA between day 7-21 after DT2, the exact time-point being based on the density of gametocytaemia as measured by Pfs25 qRT-PCR. These blood

samples will be offered to 300 mosquitoes to provide preliminary evidence on the infectivity of volunteers at these time-points.

If a planned volunteer (screened and eligible for participation) is subsequently unable to participate or becomes ineligible for enrollment at the inclusion visit, or before challenge on day 0, she/he will be replaced by another volunteer enrolled at the inclusion visit. For this purpose, two to four additional volunteers (screened and eligible for participation) will be identified and enrolled at the inclusion visit to act as back-ups.

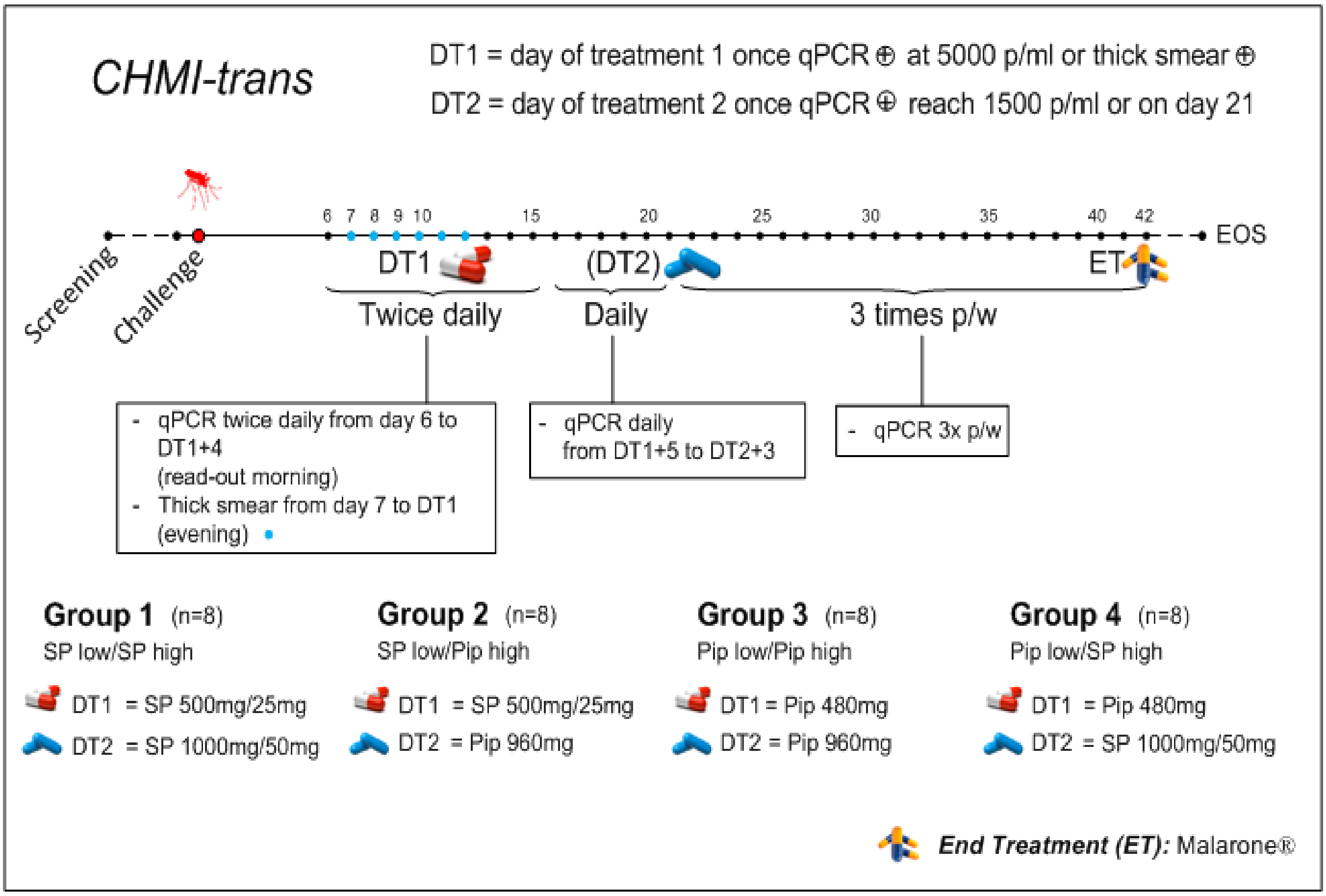

Figure 1 – Study design. After the initial challenge, data from 175[29] volunteers indicate the first possible moment of parasitaemia to be at day 6 and the first positive thick blood smear between day 7-14.5. Volunteers in this study will be monitored twice daily from day 6 onwards until malaria parasites are detected at 5000 p/mL, upon which they are treated (DT1). After DT1, volunteers will continue twice daily monitoring by 18S qPCR for another 4 days after which they will be monitored once a day for recrudescence. On day 21 or once parasites density reach 1500 p/mL, volunteers will receive a curative regimen (DT2). After DT2, volunteers will visit the study site 3 times a week until final treatment according to national guidelines with Malarone® on day 42, to assure radical clearance of all parasite stages.

4. STUDY POPULATION

4.1 Population (base)

The study population will be comprised of adult male and female healthy, malaria naïve subjects. A total of 32 subjects will be enrolled to participate in the study and be randomized to one of four treatment groups. The investigator will ensure that all subjects being considered for the study meet the following eligibility criteria. Subject eligibility is to be established and confirmed by checking through all inclusion/exclusion criteria at both screening and baseline. A relevant record (e.g. checklist) of the eligibility criteria will be stored with the source documentation at the study site.

4.2 Inclusion criteria

In order to be eligible to participate in this study, a subject must meet all of the following criteria:

1. Subject is aged ≥ 18 and ≤ 35 years and in good health.
2. Subject has adequate understanding of the procedures of the study and is able and willing (in the investigator's opinion) to comply with all study requirements.
3. Subject is willing to complete an informed consent questionnaire and is able to answer all questions correctly.
4. Subject is able to communicate well with the investigator and is available to attend all study visits, lives in proximity to the trial centre (<10 km) or (if >10km) is willing to stay in a hotel close to the trial centre during part of the study (from day 5 post-infection until DT1+4 provided that the subject has had 2 consecutive negative 18S qPCR tests (at least 24 hours apart) following DT1 treatment; or until day DT2+3).
5. The subject will remain within the Netherlands during the challenge period, will not travel to a malaria-endemic area during the study period, and is reachable (24/7) by mobile telephone throughout the entire study period.
6. Subject agrees to their general practitioner being informed and contacted about their participation in the study and agrees to sign a form to request the release by their General Practitioner (GP), and medical specialist when necessary, to the investigator(s), of any relevant medical information concerning possible contra-indications for participation in the study.
7. The subject agrees to refrain from blood donation to Sanquin or for other purposes throughout the study period and for a defined period thereafter according to current Sanquin guidelines.
8. For female subjects: subject agrees to use continuous adequate contraception** and not to breastfeed for the duration of study.

9. Subject agrees to refrain from intensive physical exercise (disproportionate to the subjects usual daily activity or exercise routine) during the malaria challenge period.
10. Subject has signed written informed consent to participate in the trial.

*(*Acceptable forms of contraception include: established use of oral, injected or implanted hormonal contraceptives; intrauterine device or intrauterine system; barrier methods (condoms or diaphragm with additional spermicide); male partner's sterilisation (with appropriate post-vasectomy documentation of absence of sperm in the ejaculate); true abstinence when this is in line with the preferred and usual lifestyle of the subject; Periodic abstinence (e.g., calendar, ovulation, symptothermal, post-ovulation methods) and withdrawal are not acceptable methods of contraception.)*

4.3 Exclusion criteria

A potential subject who meets any of the following criteria will be excluded from participation in this study:

1. Any history, or evidence at screening, of clinically significant symptoms, physical signs or abnormal laboratory values suggestive of systemic conditions, such as cardiovascular, pulmonary, renal, hepatic, neurological, dermatological, endocrine, malignant, haematological, infectious, immunodeficient, psychiatric and other disorders, which could compromise the health of the volunteer during the study or interfere with the interpretation of the study results. These include, but are not limited to, any of the following.
   - 1.1. Body weight <50 kg or Body Mass Index (BMI) <18 or >30 kg/m2 at screening.
   - 1.2. A heightened risk of cardiovascular disease, as determined by: an estimated ten year risk of fatal cardiovascular disease of ≥5% at screening, as determined by the Systematic Coronary Risk Evaluation (SCORE); history, or evidence at screening, of clinically significant arrhythmia's, prolonged QT-interval or other clinically relevant ECG abnormalities; or a positive family history of cardiac events in 1st or 2nd degree relatives <50 years old.
   - 1.3. A medical history of functional asplenia, sickle cell trait/disease, thalassaemia trait/disease or G6PD-deficiency.
   - 1.4. History of epilepsy in the period of five years prior to study onset, even if no longer on medication.
   - 1.5. Screening tests positive for Human Immunodeficiency Virus (HIV), active Hepatitis B Virus (HBV), Hepatitis C Virus (HCV)
   - 1.6. Chronic use of i) immunosuppressive drugs, ii) antibiotics, iii) or other immune modifying drugs within three months prior to study onset (inhaled and topical

corticosteroids and oral anti-histamines exempted) or expected use of such during the study period.

1.7. Any recent or current systemic therapy with an antibiotic or drug with potential anti-malarial activity (chloroquine, doxycycline, tetracycline, piperaquine, benzodiazepine, flunarizine, fluoxetine, tetracycline, azithromycin, clindamycin, erythromycin, hydroxychloroquine, etc.) (allowable timeframe for use at the Investigator's discretion).

1.8. History of malignancy of any organ system (other than localized basal cell carcinoma of the skin), treated or untreated, within the past 5 years.

1.9. Any history of treatment for severe psychiatric disease by a psychiatrist in the past year.

1.10. History of drug or alcohol abuse interfering with normal social function in the period of one year prior to study onset, positive urine toxicology test for cocaine or amphetamines at screening or at inclusion, or positive urine toxicology test for cannabis at inclusion.

2. For female subjects: positive urine pregnancy test at screening and/or at the baseline visit.
3. Any history of malaria, positive serology for *P. falciparum*, or previous participation in any malaria (vaccine) study.
4. Known hypersensitivity to or contra-indications (including co-medication) for use of sulfadoxine-pyrimethamine, piperaquine, chloroquine, Malarone®, artemether-lumefantrine, primaquine or history of severe (allergic) reactions to mosquito bites.
5. Participation in any other clinical study in the 30 days prior to the start of the study or during the study period.
6. Being an employee or student of the department of Medical Microbiology of the Radboudumc or the department of Internal Medicine.
7. Any other condition or situation that would, in the opinion of the investigator, place the subject at an unacceptable risk of injury or render the subject unable to meet the requirements of the protocol.

4.4 Sample size calculation

In this trial, we will determine the suitability of our treatment strategies to induce gametocytaemia, defined as gametocyte prevalence by Pfs25 qRT-PCR on any moment during follow-up. Based on preliminary data, we expect >95% of individuals will develop gametocytes after commencing treatment with sulphadoxine-pyrimethamine[34] or piperaquine[25]. We consider the CHMI-trans approach unsuitable for gametocyte induction

if <50% of individuals develop mature gametocytes. We therefore powered the trial to estimate a confidence interval around the proportion of gametocytaemic individuals that excludes 50%. If we include 8 individuals, retain 7 of these for the duration of follow-up (i.e. allowing for one dropout per arm), and 6/7 or 7/7 of these individuals become gametocytaemic, we will be able to estimate this proportion with a lower limit of the 90% confidence interval ≥ 54.8%.

5. TREATMENT OF SUBJECTS

5.1 Investigational product/treatments (for DT1 and DT2)

All volunteers will receive a sub-curative treatment (DT1) with either sulfadoxine-pyrimethamine or piperaquine. All volunteers will receive a curative treatment (DT2) with sulfadoxine-pyrimethamine or piperaquine on day 21 or when recrudescence of asexual parasitaemia occurs.

Volunteers treated with sulfadoxine-pyrimethamine (Fansidar®, Roche FR) will receive a subcurative dose (SP low - 500mg/25mg) and/or a curative regimen (SP high - 1000mg/50mg).

Volunteers treated with piperaquine tablets (80mg or 320mg tablet strength as piperaquine phosphate, Penn Pharmaceutical Services Limited UK) will receive a subcurative regimen (Pip low - 480 mg) and/or curative regimen (Pip high - 960 mg).

Refer to section 6 for further details regarding sulfadoxine-pyrimethamine and piperaquine.

5.2 Use of co-intervention

All volunteers will receive a final treatment (ET, end treatment) with Malarone® after CHMI as described in section 8.3.2. The following list of concomitant medications that are contraindications to Malarone® is based on the Malarone® product information sheet.

Concomitant medications:

- Use of anticoagulants (warfarin and other coumarin based anticoagulants)
- Use of Rifampicin, Rifabutin, Tetracycline or Metoclopramide
- Use of Indinavir

5.3 Escape medication (if applicable)

Volunteers may be advised to take tripelennamine crème for the local treatment of mosquito bites. Volunteers are advised to take paracetamol for complaints secondary to the CHMI (fever, muscle aches, headache, etc.). Tripelennamine crème, paracetamol or any other symptomatic treatment will be supplied to the volunteers. The maximum dose of paracetamol is 4 grams a day.

Pre-emptive rescue treatment with Malarone® can commence whenever deemed necessary by the investigator.

6. INVESTIGATIONAL PRODUCT (TREATMENT PRODUCT)

6.1 Name and description of investigational products

- **Sulfadoxine-pyrimethamine** tablets (Fansidar®, Roche FR), administered orally.
  - subcurative regimen (500mg/25mg as 1 tablet)
  - curative regimen (1000mg/50mg as 2 tablets).
- **Piperaquine** tablets (80mg or 320mg tablet strength as piperaquine phosphate, Penn Pharmaceutical Services Limited UK), administered orally.
  - subcurative regimen (480 mg, 1 tablets of 320mg and 2 tablets of 80mg)
  - curative regimen (960 mg, 3 tablets of 320mg).

6.2 Summary of findings from non-clinical studies

Please see Summary of Product Characteristics (SPC) for Fansidar® and the Investigator's Brochure for piperaquine.

6.3 Summary of findings from clinical studies

Please see Summary of Product Characteristics (SPC) for Fansidar® and the Investigator's Brochure for piperaquinefor detailed information. A short summary of the available data is given below.

6.3.1 Clinical data for sulfadoxine-pyrimethamine

A fixed-dose combination of sulfadoxine and pyrimethamine (Fansidar®), the usual successor to failing chloroquine, has been widely implemented in the last decade and is one of the most widely used antimalarial treatments in the world. Sulfadoxine-pyrimethamine (SP) has the great advantage that the entire treatment can be given as a single dose[35].

Although artemisinin-combination therapy has now replaced the use of SP throughout malaria-endemic settings, SP is still widely used as a first-line intermittent preventive treatment of malaria in pregnancy (IPTp) [36-38] and for malaria seasonal chemoprophylaxis alone or in combination with amodiaquine[39].

Sulfadoxine-pyrimethamine can effectively block two enzymes involved in the biosynthesis of folinic acid within the parasites: sulfadoxine inhibits the dihydropteroate synthetase and pyrimethamine blocks the dihydrofolate reductase. Both drugs are active predominantly against the later development stages of asexual parasites[40]. The curative regimen consists of 2-3 tablets of 500mg sulfadoxine and 25mg pyrimethamine per tablet[40].

Some studies show that treatment with SP is correlated with an increase in gametocyte carriage[41, 42].

6.3.2 Clinical data for Piperaquine

Piperaquine is a bisquinoline 4-aminoquinoline anti-malarial structurally related to chloroquine. It was synthesized independently in France and China in the 1960s[43, 44], and widely used for malaria control activities in China in the 1970's and 1980's (13). In the 1990s, piperaquine was reconsidered as a partner drug in artemisinin-based combination therapy, and the renewed development led to a novel combination formulation of dihydroartemisinin plus piperaquine, each tablet containing 40mg dihydroartemisinin and 320mg piperaquine phosphate (DHA-PQP). The mechanism of action and of resistance of piperaquine has not been well studied but is likely to be similar to those of drugs of the same class[45]. The antimalarial activity of piperaquine when administered as a single agent in a malaria challenge model has been established at QIMR Berghofer Medical Research Institute, Australia[25], in a dose finding study. Administered as a single dose (960, 640 and 480 mg) the drug rapidly cleared asexual parasitaemia. Recrudescences have occurred in 3 out of the 6 subjects treated with 480mg piperaquine, thus the contingency for a second dose of piperaquine (960 mg) in this study if this occurs[25].

6.3.3 Pharmacokinetics and Metabolism of sulfadoxine-pyrimethamine

Both sulfadoxine and pyrimethamine are readily absorbed from the gastrointestinal tract after oral administration. Both drugs are highly bound to plasma protein (about 90%). Sulfadoxine has an elimination half-life of 4.9-13.2 days and pyrimethamine, 2.1-6.5 days[35]. Peak plasma levels of sulfadoxine and pyrimethamine ranging from 51-76 mg/L are reached in approximately 4 hours (range 1.5-8 hours) and 0.13-0.4 mg/L within 2.1-77 hours, respectively, after a single tablet of 500mg/25mg[40]. Pyrimethamine has a larger volume of distribution than sulfadoxine and is concentrated in kidneys, lungs, liver and spleen. Sulfadoxine and pyrimethamine are mainly metabolized by the liver. Sulfadoxine undergoes various degrees of acetylation, hydroxylation and glucuronidation. Both drugs are excreted mainly through the kidneys[40].

6.3.4 Pharmacokinetics and Metabolism of Piperaquine

The pharmacokinetic properties of piperaquine are similar to those of chloroquine. It has a very large volume of distribution, ranging from 103 to 716 l/kg, values that are significantly larger even than comparable drugs such as chloroquine[46]. It has a very long terminal elimination half-life, 531 h (22 days) and 468 h (20 days) in adults and children, respectively[47]. The prolonged half-life results in a beneficial post-treatment prophylactic period, estimated to be about 20 days, and protecting against both *P. vivax* and *P. falciparum*. Although early recurrent infections are reduced, infections treated with DHA-PQP are more likely to produce gametocytes than artemether-lumefantrine (AL), an observation

hypothesized to reflect the lower dosing of artemisinin derivative in DHA-PQP (total ~7.5mg/kg of DHA compared to ~11.5mg/kg of artemether in AL). Furthermore, a smaller volume of distribution, and shorter half life of piperaquine is seen in children, resulting in a higher risk of recrudescence and earlier re-infection. Thus, an increase of the weight-adjusted dosage in young children may be required[48].

Piperaquine is highly lipophilic, and its oral bioavailability is approximately doubled by administration with a high-fat meal[49, 50]. However, data regarding the influence of food on the bioavailability of piperaquine in human subjects are conflicting[46, 51, 52]. In a study carried out in Papua New Guinea, a surprisingly low efficacy of DHA-PQP was reported (88% at day 42), significantly lower than that for artemether–lumefantrine (AL). However, the difference had wide confidence intervals and was apparent at day 28 but not at day 42. This reduced efficacy is in contrast to other studies carried out in Africa[53-55] and Asia[56, 57] where DHA-PQP had similar or higher efficacy to other ACTs. Because of apparent significant effects of food intake, piperaquine will be administered in a fasting state in this study.

6.3.5 Safety and tolerability of sulfadoxine-pyrimethamine

Sulfadoxine-pyrimethamine is well tolerated, with the main adverse effects reported to be gastroinstestinal disturbances, headache, dizziness and skin reactions such as photosensitivity, rash, pruritus, urticaria and slight hair loss, which are mainly associated with sulfanomides[37, 58, 59]. In very rare cases, particularly in hypersensitive patients, severe, possibly life-threatening skin reactions such as erythema multiforme, Stevens-Johnson syndrome and Lyell's syndrome may occur. The drug should be withdrawn immediately if skin reactions are observed. Furthermore, leukopenia, thrombocytopenia, megaloblastic anaemia, haemolytic anaemia, haematuria, oliguria and hepatitis have also been reported. Patients should be advised that sore throat, fever, cough, dyspnoea or purpura may be the first signs of serious side effects. The intake must be stopped immediately at the first signs of skin eruptions, a significant decrease of blood cells, or a bacterial or fungal superinfection[40].

6.3.6 Safety and tolerability of Piperaquine

Piperaquine is well tolerated both in adults and in children[47], with the main adverse events reported to be gastrointestinal disturbance such as diarrhoea[56], although this varies considerably according to geographical region. Electrocardiographic effects of piperaquine have been specifically evaluated in two studies[57, 60-62]. Both demonstrated a prolongation of the corrected QT interval during treatment (between 11 and 14ms). Very few individual

patients experienced a prolongation that could be regarded as clinically significant (>60ms); notably, QTc prolongation induced by piperaquine has not been associated with clinically relevant cardiovascular events suggesting a pro-arrythmogenic effect. Therefore, although statistically significant, the QTc prolongation observed following piperaquine therapy is unlikely to be clinically relevant. European regulatory authorities have however recommended that DHA-PQP should not be administered with food (to reduce peak concentrations), and caution that prior and post electrocardiographic monitoring should be undertaken, as well as avoidance of concomitant consumption or recent exposure to drugs at risk of QTc prolongation[61, 62].

The principal risks related to piperaquine include:

- Mild elevations in hepatic enzymes; transaminase elevations in malaria patients have typically been <2xULN, with no increases >5xULN, and with no severe liver function derangements (Hy's law cases) observed. The pattern of transaminase increases is not unusual with acute malaria, although there was a suggestion of a potentially dose-related effect.
- QTc prolongation (both QTcB and QTcF); although mostly in the range >30 msec but <60 msec, prolongations >60 msec have been observed with a single instance of QTcF that exceeded 500 msec. This risk is mitigated by administering the drug while the subject is fasting.

6.4 Drug and dose selection rationale

The rationale for the use of sulfadoxine-pyrimethamine and piperaquine as treatment is based on the fact that they are the only drugs known to increase gametocytes in patients [23, 48, 63]. Both drugs do not kill circulating gametocytes in contrast to other antimalarial drugs (e.g. artemisinins), which would not allow addressing the current study objectives.

The association between gametocyte production and transmissibility after sulfadoxine-pyrimethamine (SP) is complex. High gametocyte concentrations have been observed after treatment with SP, however, the infectivity of these gametocytes upon their appearance is lower. Possibly, because the SP treatment initiates an efflux of less mature gametocytes that require 3-7 additional days of maturation[64], and the presence of sulfadoxine-pyrimethamine in the bloodmeal of the mosquitoes[63]. Nevertheless, the considerable increase in the prevalence and density of gametocytes results in a net increase of infectivity of mosquitoes although this increase may be lower than expected based on the gametocyte concentration (the chance of mosquito infection per gametocyte is smaller).

The selection of the optimal treatment for induction of infectious gametocyte requires a thorough comparison, and possibly a combination of agents wherein the production and fitness of gametocytes are optimized. The reason to use four study arms in this study is to

directly compare the possible combinations of sulfadoxine-pyrimethamine and piperaquine, and increase the likelihood of obtaining the study-objectives.

The rationale to use suboptimal treatment (DT1) regimens is based on partial clearance of asexual parasites followed by recrudescence. This will presumably cause an increase in asexual commitment to gametocytes through terminal investment of malaria parasites under drug pressure [5, 21] [22].

In previous CHMI's[25], piperaquine was administered at single de-escalating dosages. The lowest dose used (480 mg) resulted in complete clearance of parasitaemia in 3 out of 6 subjects (cohort 3B) within 30 to 36 hours after piperaquine treatment. Recrudescent parasitaemia occurred in 3/6 volunteers which was completely cleared after a second dose of piperaquine (960 mg). Recrudescent infection was identified by routine 18S qPCR monitoring. As such, for the curative dosing regimen (DT2) and in accordance with group assignment, the higher dose (960 mg) will be given to clear recrudescent parasitaemia, without affecting (mature) gametocytes.

The rationale to use SP is based on field studies, showing an increase of transmissibility to mosquitoes after a curative treatment regimen. Therefore, a suboptimal dose of SP (500mg/25mg) is chosen to treat asexual parasites without killing (developing) gametocytes. Although the efficacy of this dose of SP on low density parasitaemia is unknown, most of the clinical data on SP are from clinical malaria cases in Africa where parasite densities are on average >1000-fold higher than in our CHMI model, we do anticipate recrudescent infections in some individuals. For the curative dosing regimen (DT2) and in accordance with group assignment, the higher dose (1000mg/50mg) will be given to clear recrudescent parasitaemia without affecting (mature) gametocytes.

6.5 Potential Risks

See Summary of Product Characteristics (SPC) for Fansidar®, the Investigator's Brochure for piperaquine, and section 6.3 Summary of findings from clinical studies above.

Based on preliminary data[25] and clinical data accumulated during previous malaria challenge studies, piperaquine was well tolerated in the treated participants. They showed a robust safety profile in the treated participants in dose of up to 960 mg when used for the treatment of uncomplicated *P. falciparum* malaria infection.

Treatment of uncomplicated malaria with sulfadoxine-pyrimethamine has been widely implemented in the last decade and has been one of the most widely used antimalarial treatments in the world with millions of doses administered. Clinical data show a strong safety profile of SP and treatment is generally very well tolerated[37, 40, 58, 59].

The risk of treating with subcurative regimens for both drugs is limited to a recrudescent infection. This risk is mitigated by the extensive follow-up (twice a day) until 4 days after DT1, and once a day after that. In case recrudescence occurs, volunteers will be treated with a curative regimen (DT2). Furthermore, in the unlikely case a second recrudescence occurs after DT2, final treatment with Malarone® will be initiated.

6.6 Potential Benefits

There are no expected benefits of sulfadoxine-pyrimethamine or piperaquine for participants participating in this study.

6.7 Risk Management

Potential risks have been identified through review of previous clinical studies conducted to date as well as review of the literature and post-marketing data for both sulfadoxine-pyrimethamine and piperaquine. Monitoring of cardiovascular effects after piperaquine treatment will be performed using a 12-lead ECG with a focus on expected maximal piperaquine concentrations after oral dosing (within 4h-12h after treatment). Healthy volunteers with a history of cardiovascular disease or clinically significant ECG abnormalities will be excluded from participation in the study, with particular attention paid to cardiac conduction.The risk to participants in this trial will be minimized in two ways:

1. Adherence to the inclusion/exclusion criteria.
2. Close clinical and laboratory monitoring to ensure the safety and wellbeing of the healthy participants.
3. Volunteers with increases in QT/QTc to >480 ms or >30 ms over baseline, after piperaquine treatment (DT1) will not receive further dosing of piperaquine during the trial. These volunteers will instead receive the alternative drug, sulfadoxine-pyrimethamine as DT2.

The overall risk to participants participating in the study is considered to be minimal and acceptable, and the potential of the results of this study to contribute towards future accelerated product development of malaria transmission blocking interventions is considered to outweigh these potential risks.

Scheduled regular clinical chemistry and haematology blood tests will also be performed, (details of time points can be seen in section 8.3.16).

6.8 Preparation and labeling of Investigational Medicinal Product

Piperaquine: Piperaquine tablets (80mg or 320mg tablet strength as piperaquine phosphate) will be supplied to Radboudumc as blister packs supplies which are manufactured and tested

for quality control purposes in accordance with Good Manufacturing Practices by Penn Pharmaceutical Services Limited in the United Kingdom. The piperaquine tablets (80mg or 320mg tablet strength as piperaquine phosphate) are packaged in blister packs. The tablet supplies will be labelled in accordance with EMA GMP requirements and the label will include information regarding identity, batch number, expiry date and storage condition.

Piperaquine tablet supplies will be held at the nominated storage condition of 15°C-25°C and protected from moisture in appropriate locked storage conditions at Radboudumc until required.

Sulfadoxine-pyrimethamine, (and ***Malarone®****)* will be acquired by Radboudumc, labelled according to identity, brand or source, and batch number. The supplies will be held in appropriate locked storage conditions at Radboudumc until required. The contents of the label for drug to be administered to the participants will be in accordance with all applicable regulatory requirements.

6.9 Drug accountability

The principal investigator must ensure that sulfadoxine/pyrimethamine and piperaquine are stored in an appropriate storage room. Accurate records must be maintained regarding the receipt of the treatments, which include: drug name, date received, lot number, amount received.

Accurate records must also be maintained regarding administration of drugs to volunteers. These records will be kept by the investigators. This includes:

- Volunteer identification number
- Date and dose of drugs dispensed
- Signature of the person administrating the drugs.

NON-INVESTIGATIONAL PRODUCT (CHALLENGE PRODUCT)

6.10 Name and description of non-investigational product(s)

P. falciparum 3D7-infected *Anopheles stephensi* mosquitoes for the purpose controlled human malaria infection.

6.11 Summary of findings from non-clinical studies

The *P. falciparum* 3D7 parasite is a clone of the NF54 strain which was isolated from a patient living in the Schiphol-area in the Netherlands. In clinical studies and *in vitro* studies, this parasite has been shown completely susceptible to multiple antimalarials, including sulfadoxine-pyrimethamine, piperaquine, chloroquine, mefloquine, atovaquone/proguanil and arthemeter/ lumefantrine (see D2c, 'Product information *Plasmodium falciparum* infected *Anopheles* mosquitoes').

6.12 Summary of findings from clinical studies

CHMIs are well accepted as a powerful tool for the evaluation of parasite development in humans. The Radboudumc has the experience and infrastructure to conduct CHMIs. Our center uses a sensitive method for parasite detection by real-time qPCR that allows detection of malaria infection in an early stage, resulting in early treatment.

There is a large clinical experience with infecting humans by the bite of *P. falciparum*-infected mosquitoes. Since 1986 more than 3,500 volunteers at global scale have had a CHMI by the bites of mosquitoes fed on cultures of *P. falciparum* gametocytes to produce *P. falciparum* sporozoites (Chulay et al., 1986). This has proven to be a reproducible, predictable and safe method of inducing *P. falciparum* malaria. The results of such studies were summarized in 1997 (Church et al., 1997), in 2007 (Epstein et al., 2007) and in 2012 (Roestenberg, de Vlas, Nieman, Sauerwein, & Hermsen, 2012). Most CHMI studies worldwide are conducted with the 3D7 clone.

The *P. falciparum* isolate NF54 and the 3D7 clone have been tested for the purpose of challenge infection in 353 volunteers in 22 CHMI studies in the Radboudumc. In these studies >98 % of malaria-naive volunteers developed patent parasitaemia after bites from 5 NF54 infected mosquitoes. The time range to blood stage parasitaemia detectable by thick smear (prepatent period) was between 7.0 and 14 days. Mild-moderate solicited adverse events were generally experienced by all study subjects, most commonly headache (95%), general malaise/fatigue (65%) and fever (90%)[29]. Gastro-intestinal complaints, including nausea, diarrhoea and abdominal pain were also reported, mainly following intake of atovaquone-proguanil. Symptoms that were severe enough to prohibit daily activities, occurred in 49% of participants.

6.13 Summary of known and potential risks and benefits

There is no benefit expected for subjects participating in this study. The risk to subjects after challenge infection with 3D7-infected mosquitoes will be minimized by adherence to the inclusion/exclusion criteria and close clinical monitoring, which ensures that subjects with malaria will be treated at earliest stages of parasitaemia. The risks of a CHMI for malaria-naïve subjects include i) discomfort induced by mosquito bites, ii) discomfort associated with periodic blood drawing and iii) risk of acquiring mild clinical *P. falciparum* malaria.

Mosquito bites are known to cause mild discomfort associated with mosquito feeding. A small degree of inflammation and pruritus typically accompanies the bite of the insect. Anaphylaxis after mosquito bites is extremely rare and has never been reported after CHMI. While significant allergic reactions are extremely rare, in the event of an allergic reaction, epinephrine, anti-histamines, on-call physician and resuscitation equipment are available on site. The Radboudumc, an established site for CHMIs, is fully equipped to manage anaphylaxis and any other medical emergency.

Frequent blood draws will be necessary to closely monitor the subjects and to perform qPCR and thick smears for detection of *P. falciparum* parasitaemia. Universal precautions will be taken for protection of the volunteer and study personnel. The total amount of blood collected will be maximally 500 ml over the trial period (of 64 days, plus up to 120 days for the screening period) in agreement with guidelines of the Sanquin blood bank.

Intensive follow-up with qPCR performed on samples taken twice daily (and thick smears once daily in the evening), will ensure early treatment - preventing high levels or prolonged duration of parasitaemia that would put the subject at undue risk. Severe/complicated malaria has never been described in a CHMI. Mild malaria symptoms include headache, myalgia, fever, chills, sweats, nausea, vomiting, and diarrhoea. Researchers at the Radboudumc have extensive experience with the care of clinical malaria.

Although subjects often become symptomatic with mild malaria after CHMI, rapid diagnosis by qPCR and/or thick smears and subsequent treatment quickly attenuates the illness so that the infection does not place the subject at undue risk.

The exposure to infected *P. falciparum* 3D7-infected mosquito bites will occur at Radboudumc insectary which has a double-door barrier system along with a double blower (negative pressure wind blockade).

6.14 Description and justification of route of administration and dosage

Study subjects will be exposed to malaria for the purpose of challenge infection through (cutaneous) bites of *P. falciparum* 3D7-infected female *Anopheles stephensi* mosquitoes. This is the natural route of infection and the one with which most experience has been

accumulated in CHMI trials. Volunteers will be exposed to the bites of 5 3D7-infected mosquitoes, which is the gold-standard dosage for CHMI studies worldwide.

6.15 Dosages, dosage modifications and method of administration

See also paragraph 7.5. Subjects receiving a malaria challenge infection with the 3D7 clone of *P. falciparum* will receive 5 infectious bites from *A. stephensi* mosquitoes infected with 3D7 sporozoites. Previous studies have demonstrated that with this dosage, near 100% of malaria-naïve volunteers develop a blood stage malaria infection (Verhage et al., 2005).

6.16 Preparation and labeling of Non Investigational Medicinal Product

The culture of parasites and infection of mosquitoes has been a routine procedure for over 20 years in the Malaria Unit of the Central Animal Facility of the Radboudumc, Nijmegen. The 3D7 parasite is a clone of the NF54 *P. falciparum* isolate, originated from the Schiphol area. The isolate was originally derived from patient material and is cultured in vitro in RPMI-1640 medium with 10% serum and 5% haematocrit red blood cells. Both the serum and the red blood cells are obtained from the Nijmegen department of the Sanquin Bloedbank region Zuid-Oost. These blood products are negative for malaria and Hepatitis B surface Anitgen (HBsAg), and seronegative for HIV, HCV, HumanT-lymphotropic Virus (HTLV) 1+2 and syphilis. The cultures are checked for bacterial, fungal and *Mycoplasma* contamination.

After 14 days of *in vitro* culture, the sexual stage parasites will be harvested for feeding to 1-5 days old laboratory cultured *A. stephensi* mosquitoes via a 'membrane feeder'. The percentage *P. falciparum*-infected mosquitoes will be assessed 6-9 days after feeding and one day prior to the CHMI. Mosquitoes will be kept in the same midi-cage from Membrane Feed for Sporozoite production until the day before CHMI. On the day before CHMI, a sample of 10 mosquitoes from each batch is checked for the presence of sporozoites. The best batch will be selected for CHMI requiring a batch of at least 40% infected mosquitoes. Mosquitoes are then transferred from midi- cages to small CHMI-cages. Each step in this process is performed by an experienced technician, and checked and recorded on standardized forms by another technician according to standard operating procedures.

6.17 Drug accountability

The date, time of collection and person collecting the mosquitoes is filled in on a standard table. This section will be signed by the responsible employee.

7. METHODS

7.1 Study parameters/endpoints

7.1.1 Main study parameter/endpoint

- Frequency and magnitude of adverse events in the CHMI-trans model in study groups.
- Prevalence of gametocytes in the CHMI-trans model in study groups.

7.1.2 Secondary study parameters/endpoints

- Peak density and time-point of peak density of gametocytes by qRT-PCR.
- The area under the curve of gametocyte density versus time.
- Assessment of the dynamics of gametocyte commitment, maturation and sex-ratio.

7.1.3 Exploratory study parameters/endpoints

- Assessment of gametocyte infectivity to *Anopheles* mosquitoes by DMFA.
- Quantification of gamete formation and fertilization as ex vivo indicators of gametocyte fitness.
  - Antibodies to proteins expressed/exported during early gametocyte development.
  - The association between the timing and density of gametocyte carriage and antibody responses to gametocyte antigens (including but not restricted to Pfs48/45, Pfs230, Pfs16, PfGEXP5).
  - Antigen specificity and/or functionality of CHMI induced antibodies against *Plasmodium falciparum.*
  - The specificity and/or functionality of CHMI induced T cell responses against *Plasmodium falciparum.*
  - Epigenetic profiling of immune cells via Chromatin Immunoprecipitation and sequencing and/or RNA transcriptome profiling through whole mRNA-sequencing, PCR or microarray.
  - Phenotype and/or function of immune responses to CHMI (with specific focus on innate lymphoid cells, ɣδT cells, monocytes, platelets, NK cells and granulocytes).
  - Hematological profiles for diagnostics in malaria

7.2 Randomisation, blinding and treatment allocation

This is an open-label study. The 32 volunteers will be allocated to one of the four groups at random according to a randomization list derived from the Microsoft Excel command ASELECTUSSEN (0,1000). Stratification will not be performed. An independent investigator at the Radboudumc, not involved in the clinical trial, is responsible for performing the

randomization and for assigning volunteers according to the randomization list. A second employee, not involved in the assignment of volunteers, will check to see if randomization is done correctly. Every volunteer number will be linked to a random number between 0 and 1000. The ten lowest numbers will be assigned group 1, the next ten lowest numbers will be assigned to group 2, the next ten to group 3, and the highest ten numbers to group 4. If two identical numbers are produced, the whole procedure is repeated. The randomization list is kept in a fireproof clinical trial cabinet at Radboudumc.

7.3 Study procedures

7.3.1 Screening period: Screening, Inclusion and Baseline visits

Volunteers who wish to participate in the trial will be asked to complete an informed consent questionnaire. Their understanding of the trial will be tested after discussing the study with the investigator during informed consent, and prior to being asked to sign and date the consent form. Volunteers who fail to answer all questions correctly on their first attempt are allowed to re-take the questionnaire following further discussion with the investigator, and provided they subsequently answer all questions correctly, they may then complete the consent form and be screened for the trial.

Subjects who sign informed consent will undergo complete screening including a medical history, physical examination, vital signs, ECG, urine tests and laboratory evaluations (see sections 8.3.9-8.3.10). If physical examination, vital signs or laboratory values are out of the normal range a repeat measurement may be obtained.

Subjects who meet the eligibility criteria will be invited back for enrollment into the study at the inclusion visit, which will occur 6 to 8 days prior to the planned challenge day (day 0). Some screening assessments, including physical examination, vital signs, urine tests and laboratory evaluations, will be repeated at this inclusion visit.

Following this screening period of up to 120 days, subjects who continue to meet the eligibility criteria (Section 4.2 and 4.3) will present to the study site the day before the challenge infection for baseline assessments. Patient history will be taken and all adverse events that have occurred since screening will be noted.Only subjects who still meet the inclusion criteria will receive bites of infected mosquito bites. For each subject, study start will be defined as the day of the inclusion visit.

7.3.2 Controlled Human Malaria Infection

Only subjects that met the inclusion criteria will undergo a malaria challenge infection. On the challenge day, all subjects will be exposed to the bites of five 3D7 *P. falciparum* infected mosquitoes. Mosquito feeding will be allowed for 10 minutes. Volunteers will receive a local

treatment (tripelennamine crème) for mosquito bites and will be observed for 15 minutes after the feed. Directly after the feed, the mosquitoes will be dissected by a technician of the mosquito unit according to standard operating procedures. Exposure will be repeated until five infected mosquitoes have fed on each volunteer.
As long as there are volunteers present in the mosquito unit, there will be supervision by one of the clinical investigators. Another clinical investigator will be on call, in case of emergency. Emergency aid kits will be present and readily available at any location, whenever there are volunteers present.
After malaria challenge infection subjects will be observed closely according to an intensive out-patient follow-up schedule including frequent safety analyses (see section 8.3.10 for details). The study design is illustrated in more detail in the flowchart in section 8.3.16.
Subjects are required to reside locally within close proximity to the study site, within the city of Nijmegen. In cases where subjects are not local, subjects will be provided accommodation at a hotel nearby from the fifth day after CHMI until DT1+4 provided that the subject has had 2 consecutive negative 18S qPCR tests (at least 24 hours apart) following DT1 treatment; or until day DT2+3. For all subjects, during this period all relevant investigations will be carried out on an outpatient basis at Radboudumc, including frequent safety analyses (section 8.3.16 for details).

From the sixth day up until four days after treatment 1 (DT1+4) post-CHMI, assessments of parasite densities using qPCR will be performed on samples collected twice daily. Read-out of qPCR will only take place once daily. Therefore, a thick blood smear analysis will be performed in the evenings from day 7 until day of treatment (DT1). Once daily assessment by qPCR will start from 5 days post treatment 1 (DT1+5) until three days after day of treatment 2 (DT2+3). From DT2+3) until day 42, subjects will be seen 3 times a week or otherwise in case of symptoms. Subjects will measure their temperature twice daily, and contact the clinical investigators when any symptoms, complaints or fever occurs. All subjects will receive a final treatment (ET) according to national guidelines with atovaquone/proguanil (Malarone®) on day 42. In case a volunteer remains 18S qPCR and Pfs25 qRT-PCR negative for 7 days after DT1, final treatment with Malarone® will also be initiated and end of study will apply for the volunteer. All subjects will visit the study site for a follow-up visit on day 1, 2 and 3 after commencing final treatment (ET+1, 2 & 3). All subjects will be seen for a final control visit on day 64 after CHMI. The study design is illustrated in more detail in the flowchart in section 8.3.16.
Day time qPCR assessment will be performed in real time while evening samples will be assessed the next morning. To maintain safety, thick smears will be performed on all subjects from day 7 until DT1 who attend for follow-up in the evening or when a subject is

febrile (≥38.0°C) after DT1+4 (after morning visits). If a thick smear is positive in the evening, treatment will be started immediately.

As soon as a qPCR or thick smear result is deemed positive for malaria parasites, the technician will inform the trial clinician. Treatment 1 (DT1) will be initiated using the following criteria:

1. One positive 18S qPCR above 5000 parasites/mL
2. One positive thick smear (which will be made if a volunteer attends an evening follow-up visit or on decision of the trial clinician).

Treatment 2 (DT2) will be initiated using the following criteria:

1. Occurrence of recrudescent infection:
   - as assessed by 18S qPCR above 1500 parasites/mL and (possibly or probably related) symptoms.
   - (in absence of symptoms) as assessed by 18S qPCR above 1500 parasites/mL and asexual parasites counts for >50% of total parasites (measured by 18S qPCR and Pfs25 qRT-PCR).
2. One positive thick smear (which will be made if a subject is febrile (≥38.0°C) after morning visit).

If treatment has to be initiated, the trial clinician will contact the volunteer for immediate treatment. Preferably, the volunteer should return immediately or at least within 1 hour. If the volunteer is not reachable by phone, his/her contact person will be called and a search is started.

During the entire study period subjects will be instructed to call the trial physicians at any time if they experience severe symptoms. The trial physician can decide to initiate additional diagnostics, clinical observation/monitoring or (symptomatic) treatment at all times.

For unexpected laboratory abnormalities, the laboratory test will be repeated. If there is any ambiguity regarding the decision to include or exclude a volunteer, the clinical supervisor will discuss the case with the local safety monitor and make the final decision after that, if necessary with consultation of a specialist. If volunteers prove to be eligible, they will be invited to the next visit.

All subjects who are exposed to *P. falciparum* will be treated. Treatment after challenge infection will be based on the above mentioned criteria. Additionally, final treatment with Malarone® (ET) can be initiated in any of the following situations:

1. By decision of study doctor or the safety monitor
2. In consultation with the cardiologist
3. On request of the volunteer

4. On day 42 post CHMI
5. When LDH > 1000 U/l

Additionally, treatment with DT1 or DT2 can be initiated in the following situation: when platelet levels are <120x 10^9/L DT1 can be initiated, and when platelet levels are <50x 10^9/L DT2 can be initiated.

7.3.3 Treatment with sulfadoxine-pyrimethamine (DT1 and DT2)

All volunteers in group 1 and 2 will be treated with a subcurative regimen (DT1) of SP (Fansidar®, 500mg/25mg). In group 1 and/or 4, a curative regimen (DT2) of SP (Fansidar®, 1000mg/50mg) will subsequently be initiated when recrudescence occurs or on day 21 post challenge infection, whichever occurs first. All volunteers will be treated based on the predetermined criteria mentioned above. During treatment, complaints of malaria infection will be treated symptomatically. In addition to specific treatment with Fansidar®, symptomatic treatment will be administered at the discretion of the attending physician.

Volunteers will not be admitted to the hospital during this study, unless the study doctors or the safety monitor deems it necessary, or on request of the volunteer.

7.3.4 Treatment with piperaquine (DT1 and DT2)

All volunteers in group 3 and 4 will be treated with a subcurative regimen (DT1) of Pip (piperaquine 480mg). In group 2 and/or 3, a curative regimen (DT2) of Pip (piperaquine 960mg) will subsequently be initiated when recrudescence occurs or on day 21 post challenge infection, whichever occurs first. All volunteers will be treated based on the predetermined criteria mentioned above.

Treatment with piperaquine will be given after a fasting period of ≥ 3 hours. If dosing is to occur in the evening, subjects will be required to fast for ≥3 hours prior to receiving treatment. Subjects will be required to fast for a further four hours anytime after dosing with piperaquine.

During treatment, complaints of malaria infection will be treated symptomatically. In addition to specific treatment with piperaquine, symptomatic treatment will be administered at the discretion of the attending physician.

Volunteers will not be admitted to the hospital during this study, unless the study doctors or the safety monitor deems it necessary, or on request of the volunteer.

7.3.5 Treatment with Malarone® (ET)

All volunteers will be treated with Malarone® (ET) on day 42. The treatment will consist of the drug Malarone® (atovaquon/proguanil). Dosing of Malarone® will be as follows: once daily 4 tablets of 250/100mg, during three days according to Dutch SWAB guidelines. During and

one day after Malarone® treatment qPCR is performed directly in collected blood samples. If qPCR remains positive after Malarone® treatment (usually the result of parasite debris remaining in the bloodstream) a thick blood smear will be performed to confirm the absence of intact malaria parasites.
Volunteers will not be admitted to the hospital during this study, unless the study doctors or the safety monitor deems it necessary, or on request of the volunteer.

7.3.6 Physical examination

A complete physical examination will include the examination of general appearance, skin, neck, eyes, throat, lungs, heart, abdomen, back and extremities, and a routine vascular and neurological examination.
Height (cm) and body weight (to the nearest 0.1 kilogram [kg] in indoor clothing, but without shoes) will also be measured, at screening only. Body mass index (BMI) will be calculated using the following formula: BMI = Body weight (kg) / [Height (m)]$_2$ and converted to an integer.

7.3.7 Vital signs

Vital signs including body temperature, blood pressure (BP) and pulse measurements will be determined and recorded at set time points during the study. Systolic and diastolic BP will be measured while the subject is sitting, with back supported and both feet placed on the floor, using an automated validated device, with an appropriately sized cuff. In case the cuff sizes available are not large enough for the subject's arm circumference, a sphygmomanometer with an appropriately sized cuff may be used.
If vital signs are out-of-range at screening or inclusion, the investigator may obtain two additional readings, so that a total of up to three consecutive assessments are made, with the subject seated quietly for approximately five minutes preceding each repeat assessment. At least the last reading must be within the normal range in order for the subject to qualify.
Temperature will be measured according to local practice, consistently throughout the study. The thermometer used should have a precision of 0.1ºC. The same route should be used throughout the study.

7.3.8 Patient-reported outcomes (study diary)

At the inclusion visit, subjects will be issued symptom diaries and thermometers. They will be asked to record all symptoms and medication use daily from the day of inclusion until end of study. The subject diary will be reviewed at each study visit and used as a basis for discussion of possible adverse events or medication use. If the occurrence of an adverse event or use of medication is confirmed by the study physician, it is recorded in the subject’s study file (see also: section 9.3).

Subjects will also be asked to measure their temperature orally every morning from the day of the malaria challenge infection until the third day of atovaquone/proguanil treatment, and record this temperature in their study diaries.
At the end of the study, the diary will be collected and kept as source data with the subject's study file.

7.3.9 Electrocardiogram

A standard 12 lead ECG will be performed at screening and 4-12 hours after treatment with piperaquine (focus on expected maximal piperaquine concentrations after oral dosing). Additional ECG assessments may be performed at anytime throughout the study at the discretion of the investigator.
All assessments will occur after the subject has rested for approximately 10 minutes in the supine position. Calibration should be performed per the local site requirements. Each ECG tracing will be labeled with the subject number and date, and kept in the source documents at the study site. Interpretation of the tracing must be made by a qualified physician and documented in the Case Report Form (CRF). Minimally, the CRF will contain date and time of ECG, heart rate, PR interval, QRS duration and QT interval (corrected). Clinically significant abnormalities will also be recorded in the CRF and reported to the Safety Monitor.

7.3.10 Blood sampling and safety laboratory evaluations

During the study, blood samples will be drawn for screening, safety and research purposes. The blood sampling schedule in the flowchart (section 8.3.16) shows when blood will be drawn. Hemoglobin, hematocrit, red blood cell count, white blood cell count with differential (e.g. neutrophils, basophils, eosinophils, monocytes, lymphocytes) and platelet count will be measured at regular time points during the study.
Alkaline phosphatase, total bilirubin, creatinine, γGT, LDH, potassium, AST, ALT, sodium, highly sensitive troponin T and urea will be measured at regular time points during the study. Glucose, triglycerides and cholesterol will be measured only at screening.
In the event that an asymptomatic individual has evidence of an elevated troponin level, a second sample may be obtained to discern whether the result could represent a false positive (0.4% of tests).
A midstream urine sample (approx. 30 ml) will be obtained at screening, inclusion and, for female subjects only, at baseline. In this sample the presence of amphetamines and cocaine will be assessed; the sample taken at inclusion will also be tested for the presence of cannabis. Additionally, for female participants a commercially available hCG urine test will be used to test for pregnancy at screening and baseline.

In the case where a laboratory assessment is outside the reference range for the laboratory at screening and/or inclusion, a decision regarding whether the result is of clinical significance or not shall be made by the clinical investigator and shall be based, in part, upon the nature and degree of the observed abnormality. The assessment may be repeated once prior to randomization.

In all cases, the investigator must document in the source documents, the clinical considerations (i.e., result was/was not clinically significant and/or medically relevant) in allowing or disallowing the subject to continue in the study.

7.3.11 Analysis of asexual parasite densities after challenge infection

qPCR for assessment of parasite densities will be performed directly in volunteer samples, as discussed previously (see section 8.3.2).

qPCR is performed according to a standard procedure as previously described (Hermsen et al., 2001) with small adjustments. In short, qPCR will be performed on the multicopy 18S ribosomal RNA gene. All samples are spiked with the extraction control Phocine Herpes Virus (PhHV) to determine efficacy of DNA isolation.

Thick smears will be performed directly in volunteer samples, as discussed previously and if deemed necessary by the clinical investigator (see section 8.3.2), according to a standard operating procedure which is based on an internationally harmonized protocol for thick smears in CHMIs (Moorthy et al., 1998) and (WHO, 2010). Per slide, the number of fields correlating to 0.5 µl of blood will be read. Slides are considered positive if they contain 2 or more parasites in these fields.

7.3.12 Direct Membrane Feeding Assays (DMFA)

A reproducible *CHMI-trans* model requires sufficient densities of circulating mature gametocytes in subjects to ensure the generation of adequate numbers of infected *Anopheles* mosquitoes after blood feeding, either directly on the skin (DSF) or through a membrane-covered device that contains a venous blood sample with gametocytes (DMFA).

Following treatment with sulfadoxine-pyrimethamine and/or piperaquine, transmission studies will be undertaken when gametocytaemia appears. To evaluate infectivity in vector mosquitoes we will use a direct membrane feeding assay (DMFA). This study will use approximately 300 female mosquitoes for DMFA per time-point to maximize the precision of mosquito infection estimates at low infection rates.

Blood will be collected (see flowchart, section 8.3.16) from each participant for membrane feeding assays with *An. stephensi* mosquitoes. For membrane feeding studies, blood will be kept at 38°C (to prevent premature exflagellation) for up to 35 minutes until dispensed into

membrane feeders. The experimental infection of mosquitoes by DMFA will be performed up to 3 times prior to curative anti-malarial treatment at the End of Study with Malarone®.

For the DMFA, female mosquitoes (3-6 days old) will be distributed into 1L plastic containers with gauze lids (~300 females/container/time point) and starved overnight prior to feeding on *P. falciparum*-infected blood samples. Mosquitoes will be allowed to feed on the blood through parafilm membranes attached to water jacketed glass feeders attached to a 39°C water bath. Mosquitoes will be allowed to feed for up to 30 min in the dark. In preliminary experiments, we have achieved 90% feeding rate with our *Anopheles stephensi* colony. Non-engorged mosquitoes will be identified and discarded. After blood feeding, mosquitoes will be maintained in environmental chambers set at 26°C and provided with 5% glucose as described[65].

Six to nine days after blood feeding, mosquitoes will be dissected and examined for oocysts in midgut preparations. For permanent preparations, oocysts will be stained with 1% mercurochrome in H2O for 5 to 60 minutes then fixed in 1% glutaraldehyde or formaldehyde[66]. Oocysts will be counted per mosquito dissected and recorded. Relationship between parasitaemia, gametocytaemia and mosquito infection (both oocyst prevalence and intensity) will be determined using generalized-linear mixed models[67]. The number of mosquitoes dying prior to dissection will be recorded. For mosquitoes that cannot be examined by microscopy due to logistical reasons (e.g. if multiple DMFA experiments are conducted on the same day, the number of available mosquitoes may exceed the maximum number that can be dissected), infection status will be determined on day 10 following the blood meal by circumsporozoite ELISA, followed by PCR confirmation based on the 18S rRNA target[68]. At this day, ELISA-based detection of malaria parasites has equal sensitivity compared to microscopy or PCR[68, 69].

7.3.13 Quantification of gametocytes, gametocyte sex ratio and gametocyte infectivity.

A detailed quantification of gametocytes, gametocyte sex ratio, and gametocyte infectivity will be assessed by using molecular markers, and ex vivo assessments of gametocyte fitness after CHMI challenge infection. Quantification of gametocytes will be performed using a Pfs25 mRNA QT-NASBA, and quantitative reverse transcription (qRT)-PCR[31, 70] P, targeting PfGEXP5[71], PF14_0748 (young gametocytes)[72] PF14_0367 (mature gametocytes)[72] and P230p (male gametocytes)[31]. Additional markers of gametocyte maturity and sex ratio will be incorporated as these become available.

qRT-PCR determination of circulating gametocytes will be performed on every day after detection of parasitaemia by 18S qPCR.

Blood samples will be taken from EDTA-vials taken for routine qPCR analysis (see flowchart, section 8.3.16). 100μL will be directly transferred into a cryovial containing 400μL of RNAprotect for qPCR stage composition analysis. Another 1mL will be enriched prior to analysis, through gametocyte separation by magnetic sorting. This approach takes advantage of the differential deposition of paramagnetic hemozoin by different parasite stages: asexual ring stages have very low hemozoin content, while later stages including all gametocyte stages have higher content; therefore this method allows for the enrichment of gametocytes using magnetic fractionation[73, 74]. In both samples, gametocyte presence will be determined using an established multiplex qRT-PCR stage composition assay that targets gametocyte specific RNA and detects both immature gametocytes and mature gametocytes [72]. Furthermore, thick smears will be analysed on DMFA time-points to look for gametocytes (see 8.3.16 Flowchart Study Design).

7.3.14 Immunological assays

Blood samples will be taken for isolation of peripheral blood mononuclear cells (PBMCs) and serum (see flowchart 8.3.16).

PBMCs and sera will be frozen and can then be used by the Radboudumc or its collaborators for exploratory immunological assays to further analyse the phenotype or functionality of the immune response during and after malaria infection.

In order to assess (antigen specific) T cell responses, the HLA-type of volunteers may be determined.

Antibody responses will also be measured to gametocyte antigens, including recombinant proteins Pfs48/45 and Pfs230 using standard ELISA methodologies[75].

7.3.15 Case report forms and data collection

All data collected by the investigators is registered in electronic case report forms. The investigator’s notes are collected in subject study files and are considered source data. Since all subjects will be healthy, there is no medical file for the study subjects, with exception of the medical file in case of adverse events/reactions resulting in a medical consultation or hospitalization. In this case the medical file will also be considered as the source data. The diaries, produced by the study volunteers are also considered source data. They will be kept as source documents together with the investigator’s notes.

7.3.16 Flowchart Study Design

	Screening	Inclusion	Base line	CHMI	Phase 1			Phase 2			Phase 3			EOS
Visit Number	V1	V2	V3	V4	V5-24			V25-30				V41	V42-44	V45
Trial timeline	-120 to -11	-8 to -6	-1	0	6-15	DT1[9]	DT1+1-4	16-21	DT2[10]	DT2+1-3	DT2+4-40	42 (ET)	ET+1-3	64
Number of visits	1x	1x	1x	1x	2x/day	2x/day	2x/day	1x	1x	1x	3x/week	1x	1x	1x
Informed consent	X													
Eligibility criteria	X		X											
Demographic data, Medical history	X													
Physical examination and vital signs	X	X			X[11]	X[11]	X[11]	X[11]	X[11]	X[11]	X[11]	X[11]	X[11]	
ECG	X					X[12]			X[12]					
Temperature	X	X	X		X	X	X	X	X	X	X	X	X	X
Challenge with 5 infected mosquitoes				X										
Collecting (serious) adverse events	as necessary[17]													
Subcurative treatment[1]						X								
Curative treatment[2]									X					
Final treatment (ET; Malarone®)												X		
Haematology tests[3] (2.0ml)	X	X			X	X	X	X	X	X	X	X	X	X
Biochemistry tests[4] (3.0ml)	X	X					X[13]			X[13]			X[13]	X
HsTropT + LDH (2.0ml)					X	X	X	X	X	X	X	X	X	
Serology (5.0ml)[5]	X	X												
Pregnancy and toxicology urine test[16]	X	X	X											
Parasitology[6] (qPCR)			X		X	X	X	X	X	X	X	X	X	X
Parasitology (thick blood smear)					X[14]				X[14]		X[14]			
Parasitology[7] (qRT-PCR)			X				X	X	X	X	X	X	X	X
DMFA									X		X[15]			
Exploratory immunology			X		X	X	X		X	X	X			X
Safety report[8]							X							X
Total blood volume collect[18]	12ml	8ml	61ml		114ml	9ml	38ml	35ml	19ml	30ml	80ml	7ml	22ml	65ml

1 Sulfadoxine-pyrimethamine (500mg/25mg) or piperaquine (480mg) according to group assignment for DT1
2 Sulfadoxine-pyrimethamine (1000mg/75mg) or piperaquine (960mg) according to group assignment for DT2
3 Hemoglobin, hematocrit, platelets, red blood cell count, white blood cell count + differentiation
4 Creatinine, urea, sodium, potassium, bilirubin, AF, γGT, AST, ALT, LDH and hs-Trop-T. Additional at screening: cholesterol, triglyceride + glucose
5 HIV, HBV, HCV, *P. falciparum(screening only)*
6 18S qPCR for blood stage *P. falciparum*. Additional thick blood smear only on indication, blood will be taken from qPCR samples.
7 Pfs25 qRT-PCR for sexual stage *P. falciparum.*
8 A safety report will be compiled upon all subjects having completed day 21 after challenge and at the end of the study.
9 After one positive 18S qPCR > 5000 p/ml for *P. falciparum* or one positive thick blood smear.
10 When recrudescence occurs; one 18S qPCR > 1500 p/ml for *P. falciparum* or one positive thick blood smear, or on day 21, whichever comes first
11 On indication. Performed at treatment visit only when volunteers are qPCR positive.
12 Within 4-12 hours after treatment with piperaquine
13 On DT1+2, DT2+2 and ET+2

[14] On evening visits from day 7 until DT1, and at DMFA timepoints. Additional thick blood smear only on indication, blood will be taken from qPCR samples.
[15] On day 4 after DT2 and between day 7-21 after DT2 (based on the height of gametocytaemia as measured by Pfs25 qRT-PCR)
[16] Toxicology urine tests at screening and inclusion visits only. Pregnancy test for female subjects at screening and baseline visits only,
[17] Only SAE's that are possibly or probably related to trial will be collected prior to inclusion.
[18] Total blood volume: 500ml

7.4 Withdrawal of individual subjects

Subjects can leave the study at any time for any reason if they wish to do so without any penalty or loss of medical benefits.

The investigator can decide to withdraw a subject from the study for urgent medical reasons.

Volunteers can be withdrawn from the study procedures at the discretion of the clinical investigator or the local safety monitor for the following reasons:

- Any serious adverse event
- Any adverse event that, according to clinical judgment of the investigator, is considered as a definite contraindication to proceeding with the study procedures.
- The use of concomitant, chronic medication active on the immune system (e.g. steroids, immunosuppressive agents) or with known antimalarial activity against *P. falciparum*
- Pregnancy
- Withdrawal of informed consent by volunteer
- Completely lost to follow-up
- Ineligibility (either arising during the study or retrospectively, having been overlooked at screening).
- If, on balance, the investigator or safety monitor believes that continuation would be detrimental to the subject's well-being
- Volunteer non-compliance with study requirements.
- Any other protocol deviation that results in a significant risk to the subject's safety

If a subject withdrawal occurs for any reason, the investigator must make every effort to determine the primary reason for a subject's withdrawal from the study and record this information in the study file. However, in accordance with the principles of the current version of the Declaration of Helsinki, a subject does have the right to withdraw from the study at any time and for any reason, and is not obliged to give his or her reasons for doing so.

If it is felt that inclusion of the study subject's data for analysis is compromised, the subject will be terminated from the study and data will not be included in analysis. This does not preclude the ethical responsibility of the investigators to ensure the safety of the subject and ensure they receive curative therapy for malaria, and follow the subject for cardiac manifestations of disease.. All data generated before withdrawal will be included in final study analysis. Blood samples collected before withdrawal will be used/stored unless the subject specifically requests otherwise.

For subjects who are lost to follow-up (i.e. those subjects whose status is unclear because they fail to appear for study visits without stating an intention to withdraw), extensive effort (i.e. documented phone calls and e-mails) will be undertaken to locate or recall him or at

least to determine his health status. The investigator should show "due diligence" by documenting in the source documents steps taken to contact the subject, e.g. dates of telephone calls, registered letters, etc.

7.5 Replacement of individual subjects after withdrawal

If a subject withdraws before or on inclusion day he/she will be replaced with an alternate volunteer who passed screening, if possible.

7.6 Follow-up of subjects withdrawn from treatment

In the event that a volunteer discontinues the study for any reason, he/she will be required to complete all safety follow-up as appropriate, as determined by the principal investigator. All volunteers who have been exposed to the bites of infectious mosquitoes are required to take a curative regimen of Malarone® (or alternative effective anti-malarial treatment should Malarone® be contra-indicated, at the discretion of the clinical investigator).

7.7 Premature termination of the study

The study may be discontinued by the sponsor:

- On advice of the safety monitor
- On advice of the Safety Monitoring Committee (SMC)
- On advice of the clinical investigator
- On advice of the CCMO

The safety monitor, SMC, CCMO or investigators may decide to put the study on hold based on adverse events, pending discussion with the Sponsor / SMC / CCMO / safety monitor / investigators. Following discussion, it may be decided to terminate the study. Safety reporting procedures are described in section 9.

8. SAFETY REPORTING

8.1 Temporary halt for reasons of subject safety

In accordance to section 10, subsection 4, of the WMO, the sponsor will suspend the study if there is sufficient ground that continuation of the study will jeopardise subject health or safety. The sponsor will notify the CCMO without undue delay of a temporary halt including the reason for such an action. The study will be suspended pending further review by the accredited METC, except insofar as suspension would jeopardise the subject's health. The investigator will take care that all subjects are kept informed.

PATH REC and WIRB will also be notified of any decisions to prematurely suspend or terminate the study.

8.2 AEs, SAEs and SUSARs

8.2.1 Adverse events (AEs)

Adverse events are defined as any undesirable experience occurring to a subject during the study, whether or not considered related to a trial procedure or the experimental intervention. All adverse events reported spontaneously by the subject or observed by the investigator or his or her staff will be recorded.

Abnormal laboratory findings (e.g. clinical chemistry or haematology) or other abnormal assessments that are judged by the investigator to be clinically significant will be recorded as AEs (or SAEs if they meet the definition). The investigator will exercise his or her medical and scientific judgement in deciding whether an abnormal laboratory finding or other abnormal assessment is clinically significant.

If there are any severe complaints not typical for malaria infection, such as chest pain, the volunteer will be evaluated immediately by a qualified clinician using the appropriate clinical assessments (e.g. ECG or measurement of cardiac enzymes) according to standard hospital care.

8.2.2 Serious adverse events (SAEs)

A serious adverse event is any untoward medical occurrence or effect that

- results in death;
- is life threatening (at the time of the event);
- requires hospitalisation or prolongation of existing inpatients' hospitalisation;
- results in persistent or significant disability or incapacity;
- is a congenital anomaly or birth defect; or

- any other important medical event that did not result in any of the outcomes listed above due to medical or surgical intervention but could have been, based upon appropriate judgement by the investigator.

An elective hospital admission will not be considered as a serious adverse event.

The investigator will report all SAEs to the sponsor and the safety monitor without undue delay after obtaining knowledge of the events. All SAEs will be reported through the web portal *ToetsingOnline* to the CCMO, within 7 days for SAEs that result in death or are life threatening followed by a period of maximum of 8 days to complete the initial preliminary report. All other SAEs will be reported within a period of maximum 15 days after the sponsor has first knowledge of the serious adverse events.

8.2.3 Suspected unexpected serious adverse reactions (SUSARs)

Adverse reactions are all untoward and unintended responses to an investigational product related to any dose administered.

Unexpected adverse reactions are SUSARs if the following three conditions are met:

1. the event must be serious (see chapter 9.2.2);
2. there must be a certain degree of probability that the event is a harmful and an undesirable reaction to the medicinal product under investigation, regardless of the administered dose;
3. the adverse reaction must be unexpected, that is to say, the nature and severity of the adverse reaction are not in agreement with the product information as recorded in the Summary of Product Characteristics (SPC).

The sponsor will report expedited all SUSARs through the web portal *ToetsingOnline* to the CCMO. The expedited reporting of SUSARs through the web portal *ToetsingOnline* is sufficient as notification to the competent authority. The expedited reporting will occur not later than 15 days after the sponsor has first knowledge of the adverse reactions. For fatal or life threatening cases the term will be maximal 7 days for a preliminary report with another 8 days for completion of the report.

As this is an open label study in which the sponsor, investigator and the SMC are not blinded to treatment allocation, the code would not have to be broken in the case of a SUSAR.

Any SAEs, SUSARs or AEs that suggest the research places subjects or others at a greater risk of harm (including physical, psychological, economic, or social harm) than was

previously known or recognized, or that require a change to the protocol or consent will be reported to PATH REC and/or WIRB in accordance with the reporting requirements of each.

8.3 Follow-up of (serious) adverse events

8.3.1 Adverse event data collection

Safety assessments will be performed and recorded by the investigators. All adverse events/reactions (solicited and unsolicited), noted by the investigators will be accurately documented in the case report form by the investigators. For each event/reaction the following details will be recorded:

1. Description of the event(s)/reaction(s)
2. Date and time of occurrence
3. Duration
4. Intensity
5. Relationship with the intervention
6. Action taken, including treatment
7. Outcome

In addition, symptoms will be ranked as (1) mild, (2) moderate, or (3) severe, depending on their intensity according to the following scale:

- Mild (grade 1): awareness of symptoms that are easily tolerated and do not interfere with usual daily activity
- Moderate (grade 2): discomfort that interferes with or limits usual daily activity
- Severe (grade 3): disabling, with subsequent inability to perform usual daily activity, resulting in absence or required bed rest

If an AE changes in intensity during the specified reporting period, a new description of the AE will be added.

When an AE/SAE occurs, it is the responsibility of the investigators to review all documentation (e.g. hospital progress notes, laboratory, and diagnostics reports) related to the event. The investigators will then record all relevant information regarding an AE/SAE on the CRF or SAE Report Form, respectively. The investigator will attempt to establish a diagnosis of the event based on signs, symptoms, and/or other clinical information. In such cases, the diagnosis should be documented as the AE/SAE and not the individual signs/symptoms.

8.3.2 Assessment of causality

The investigators are obligated to assess the relationship between study procedures and the occurrence of each AE/SAE. The investigators will use clinical judgment to determine the

relationship. Alternative causes, such as natural history of the underlying diseases, concomitant therapy, other risk factors and the temporal relationship of the event to the challenge will be considered and investigated. The relationship of the adverse event with the study procedures will be categorized as:

Probable	An adverse event that follows a reasonable temporal sequence from the study procedure and cannot be reasonably explained by the known characteristics of the subject's clinical state.
Possible	An adverse event for which insufficient information exists to exclude that the event is related to the study procedure.
Not related	An event for which sufficient information exists to indicate that the aetiology is unrelated either because of the temporal sequence of events or because of the subject's clinical state or other therapies.

8.3.3 Follow-up of adverse events

All AEs will be followed until they have abated, or until a stable situation has been reached. AEs that result in a subject's withdrawal from the study or that are present at the end of the study will be followed up (if the volunteer consents to this) until a satisfactory resolution or stabilisation occurs, or until a non-study related causality is assigned.

Depending on the event, follow up may require additional tests or medical procedures as indicated, and/or referral to the general physician or a medical specialist.

AEs and SAEs will be reported until end of study within the Netherlands, defined as the last patient visit.

8.4 Local Safety Monitor (LSM) and Safety Monitoring Committee (SMC)

8.4.1 Local safety monitor

For this study, a local safety monitor will appointed, who is based in the Raboudumc and will be involved in the review of severe and serious adverse events and volunteer safety. He/she is an experienced clinician qualified to evaluate safety data from clinical studies with malaria infections. He/she is independent of the sponsor and the investigators.

8.4.2 Safety Monitoring Committee (SMC)

An independent Safety Monitoring Committee (SMC) will be appointed, including at least 3 individuals. Their main responsibility will be assessing any severe or serious adverse events and, if necessary, halting further study procedures. A safety report including a list of all reported adverse events and any safety laboratory values outside the normal range will be

prepared for all groups upon day 21 by all subjects and at the end of the study. For further details see section 8.3.16.

The advice(s) of the SMC will only be sent to the sponsor of the study and the funding partner, PATH. Should the sponsor decide not to fully implement the advice of the SMC, the sponsor will send the advice to the CCMO, including a note to substantiate why (part of) the advice of the SMC will not be followed.

8.4.3 Review of safety data by the safety monitor and SMC

A safety report including a list of all reported adverse events and any safety laboratory values outside the normal range will be prepared upon all subjects having completed the day 21 visit after the malaria challenge infection and at the end of the study.

These reports will be prepared by a clinical investigator and sent to the safety monitor,all clinical investigators involved and the funding partner, PATH. The safety monitor will review the safety data within 2 workdays and if warranted instruct the site to take appropriate action.

In addition, safety data for all participants will be assessed by the SMC at the end of the study. Responsibilities of the SMC are described in the SMC Charter. The advice(s) of the SMC will only be communicated to the CCMO when the sponsor does not follow this. With this notification a statement will be included indicating whether the advice will be followed.

Any highly sensitive troponin T value greater than 60ng/l will be reported to the safety monitor within 24 hours. Any safety laboratory values that lead to immediate malaria treatment will be reported to the safety monitor within 24 hours.

8.4.4 Safety stopping rules

The study may be placed on safety hold for the following reasons:

- On advice of the safety monitor
- On advice of the Principal/Clinical investigators
- On advice of the SMC
- On advice of the CCMO
- One or more participants experience a SAE that is determined to be related to the study intervention
- Two or more grade 3 adverse events in the same group of subjects, which are unexpected and possibly, probably or definitively related to the study intervention.
- Any clinical cardiac event that does not meet the criteria of SAE

The safety monitor, CCMO, or investigators may decide to put the study on hold based on adverse events, pending discussion with the safety monitor, CCMO, and investigators. In addition, the PI can always decide based on characteristics, duration and severity of

signs/symptoms to treat and stop the trial for individual cases. The PI will identify when stopping rule criteria are met and alert the appropriate parties. The safety monitor will review all available safety data on a pre-defined time point after the challenge period. If the CCMO has recommended safety hold, re-initiation of the study will require CCMO concurrence. The CCMO, PATH REC and WIRB will be informed of a safety hold by the sponsor. Following discussion, it may be decided to terminate the study.

9. STATISTICAL ANALYSIS

9.1 Primary study parameter(s)

All adverse events for each volunteer will be tabulated. Adverse events will be analyzed by calculating the proportion of volunteers in each group who report mild, moderate or severe adverse events. The frequency of signs and symptoms will be compared between groups with the chi-square test.

Any clinically important deviations from normal occurring in routine laboratory test results and/or vital signs as determined by the investigator will be listed.

Clinical laboratory data (haematology, blood chemistry, and urinalysis) which is outside of the normal range will be listed in tables. Isolated laboratory abnormalities will be reported as AEs if they are considered to be clinically significant by the Investigator. Vital signs which are outside of the normal range and clinically significant will also be listed in tables. All adverse events will be listed by participant and will include details of the treatment received prior to onset, onset time, duration, severity and relationship to the study drug.

In this trial, we will determine the suitability of our treatment strategies to induce gametocytaemia, defined as gametocyte prevalence by Pfs25 qRT-PCR on any moment during follow-up. Based on preliminary data, we expect >95% of individuals will develop gametocytes after commencing treatment with sulphadoxine-pyrimethamine[34] or piperaquine[25]. Conservatively, we consider the CHMI-trans approach unsuitable for gametocyte induction if <50% of individuals develop mature gametocytes. We therefore powered the trial to estimate a confidence interval around the proportion of gametocytaemic individuals that excludes 50%. If we include 8 individuals, retain 7 of these for the duration of follow-up (i.e. allowing for one dropout per arm), and 6/7 or 7/7 of these individuals become gametocytaemic, we will be able to estimate this proportion with a lower limit of the 90% confidence interval ≥ 54.8%.

9.2 Secondary study parameter(s)

Demographic data will be summarized by descriptive statistics and will include total number of observations (n), mean, standard deviation (SD) and range for continuous variables and number and % with characteristics for dichotomous variables.

9.3 Other study parameters

In immunological analyses we will assess differences by comparing mean values between groups or time points using either a two-tailed student's t-test (if comparing two groups) or a one-way ANOVA (if comparing more than two groups) or non-parametric equivalents. Paired tests will be used if pre-intervention values are compared with post intervention values, unpaired tests will be used if comparisons are made between groups. For discrete variables (e.g. the number of positive assays), the chi-squared test or Fisher's exact test will be used (two-tailed).

10. ETHICAL CONSIDERATIONS

10.1 Regulation statement

This study will be conducted in accordance with the latest Fortaleza revision of the Declaration of Helsinki (2013), the Medical Research Involving Human Subjects Act (WMO), the ICH Good Clinical Practice, and local regulatory requirements.

The investigators are responsible for obtaining all relevant Ethics Committee (EC) / Institutional Review Board (IRB) approvals, including WIRB approval, of the protocol and any subsequent amendments in compliance with local law before the start of the study.

10.2 Recruitment and consent

As soon as the study is approved by the CCMO and WIRB, healthy volunteers will be recruited to participate in the study. Advertisements will be placed in prominent places on university campuses and other public places as well as on the intranet of the Radboud University. Furthermore, a Facebook page (link: http://www.facebook.com/malariavaccin) showing the advertisement text will be designed to inform people about the trial. This brief advertisement will indicate a telephone number to call and an e-mail address for contact to request further information. It will furthermore indicate a website which contains a link to a short online form. This short online form requests the interested volunteer to provide their contact details and answer some initial general health and medical questions. When seemingly suitable volunteers contact investigators via e-mail, telephone or the online form, they will be invited to an information meeting during which the study will be explained to them by the study investigator. Directly after the meeting they will be provided with documents to review at home (the information sheet, the informed consent form, the application form, and the insurance text). During and after the meeting there will be time for questions. After this free discussion with the investigator, and any follow-up discussion if necessary, the volunteer will be given sufficient time to consider participation.

Volunteers who are interested in participating will be asked to fill in the application form and will be invited to come for a screening visit. Eligible subjects may only be included in the study after providing written, CCMO and WIRB-approved informed consent. Informed consent must be obtained after the informed consent questionnaire and before conducting any study-specific procedures (i.e. any of the procedures described in the protocol, including screening procedures). The process of obtaining informed consent should be documented in the subject source documents. During the screening visit, the informed consent questionnaire and health questionnaire answers will be discussed, and inclusion and exclusion criteria will be checked. Also, a letter for the general practitioner will be signed and sent after screening. Again, the investigator will answer all questions the volunteer has. The investigator will emphasise that participation in the study is entirely voluntary and that the subject may withdraw at any time, without any obligation to declare their reason for

withdrawal. However if the volunteer has undergone CHMI and not completed a course of appropriate antimalarial therapy then the volunteer will need to maintain contact with the investigators for monitoring and treatment.

The investigators will be responsible for providing adequate verbal and written information regarding the objectives and procedures of the study, the potential risks involved and the obligations of the volunteers. Volunteers will be informed that they will not gain health benefits from this study. Trainees or other students who might be dependent on the investigators or the study group will not be included in the study.

10.3 Benefits and risks assessment, group relatedness

Two major areas of ethical concern are contained within this proposal, namely the use of blood from humans and the use of human volunteers for *P. falciparum* CHMI. All partners in this proposal are aware of and follow the relevant national and international rules and regulations as they pertain to access to material of human origin and clinical research. International agreements such as the Declaration of Helsinki will be observed and respected.

10.3.1 Ethical aspects concerning the production of *P. falciparum* infected mosquitoes

The human blood and serum used for parasite culture is obtained from screened healthy blood donors from Sanquin. Continuous culture of the drug sensitive *P. falciparum* strain NF54 has been routine for the Central Animal Facility of the Radboudumc over the past 3 decades. All culture material is checked for bacterial contamination, including Mycoplasma, and for blood-borne pathogens (HIV, HBV, HCV, HTLV 1+2). The process has become highly standardized as described in standard operating procedures and was positively reviewed by an external auditor in 2014.

10.3.2 Ethical aspects concerning the use of human volunteers

Infection of humans with malaria has been carried out for nearly a century, including for therapeutic use as treatment for neurosyphilis and later for drug and vaccine evaluation. The ability to carry out this type of work is largely based on the relatively low morbidity and the lack of mortality seen in these studies since the advent of feeding mosquitoes on *P. falciparum* gametocyte cultures in 1986 [27]. The occurrence of three cardiac events in volunteers participating in phase I/IIa malaria vaccine trials in Nijmegen raised intense discussion about the safety of malaria challenge trials with respect to cardiac events. Based on recommendations of the CCMO and an External Scientific Advisory Committee to the European Malaria Vaccine Development Association, this malaria challenge trial protocol has been adjusted (see also section 1.4).

Testing in human subjects remains the only reliable and convincing way to obtain information on the immunological responses that are important for protection against malaria. Of course, the compelling need for a malaria vaccines and treatments needs to be balanced with the potential risks and discomforts of the volunteers. Explorative studies looking for new or complementary transmission blocking drugs or vaccines are of paramount importance with the potential of large-scale application in endemic countries. These transmission-blocking interventions are considered essential tools to consolidate recent gains of malaria control in recent years and move towards malaria elimination.

The study will be undertaken in accordance with Good Clinical Practices (GCP), according to the standards defined in the EEC directive 91/507/EEC, and in the Directive on Good Clinical Practice in Clinical Trials (ICH GCP, 75/318/EEC, January 1997) and under the principles of the Declaration of Helsinki; ethical permission will be sought from the CCMO the Netherlands and from the funding partner's designated IRB, WIRB..

10.4 Compensation for injury

The sponsor/investigator has a liability insurance which is in accordance with article 7 of the WMO.

The sponsor (also) has an insurance which is in accordance with the legal requirements in the Netherlands (Article 7 WMO). This insurance provides cover for damage to research subjects through injury or death caused by the study.

1. € 650.000,-- (i.e. six hundred and fifty thousand Euro) for death or injury for each subject who participates in the research;
2. € 5.000.000,-- (i.e. five million Euro) for death or injury for all subjects who participate in the research;
3. € 7.500.000,-- (i.e. seven million five hundred thousand Euro) for the total damage incurred by the organisation for all damage disclosed by scientific research for the sponsor as 'verrichter' in the meaning of said Act in each year of insurance coverage.

The insurance applies to the damage that becomes apparent during the study or within 4 years after the end of the study.

10.5 Incentives

Enrolled and challenged volunteers will receive up to 1600,- Euros in compensation for their time and for the inconveniences of taking part in this study. This is based on compensation fees for procedures as below:

- Inconvenience of blood tests and/or visits: 20,- Euros per blood sampling and/or visit

- Mosquito Challenge: 125,- Euros
- Illness and treatments compensation: 400,- Euros

Travel expenses will not be additionally reimbursed, and compensation will not be provided to volunteers who are not enrolled i.e. screen failures. Eligible volunteers who are enrolled at the inclusion visit as back-ups, but who are not challenged on Day 0 will be compensated 50,- Euros. These compensation amounts are reasonable and in line with Dutch common practice. In case of unexpected medical complications, there will be access to state-of-the-art medical treatment with full costs covered by the insurance of Radboudumc.

11. ADMINISTRATIVE ASPECTS, MONITORING AND PUBLICATION

11.1 Handling and storage of data and documents

11.1.1 Confidentiality

All parties agree to adhere to the principles of medical confidentiality in relation to clinical study subjects involved in this trial, and shall not disclose the identity of subjects to third parties without prior written consent of the subject.

All data will be anonymised; volunteer data will be identified by a unique study number in the CRF. Separate confidential files containing identifiable information will be stored in secured locations. All plasma samples, or other biological samples, with exception of those taken for safety diagnostics, will be labelled with the volunteer study identification number. Samples taken for safety diagnostic (processed by the central clinical laboratory of the Radboudumc) will be labelled with part of the subject identification code, study identification name and a fictitious birth date (only using the subjects actual birth year). The samples will not be labelled with volunteer names or actual birth dates.

The subject identification code will be kept by the principal investigator.

The investigator will maintain and retain appropriate medical and research records and essential documents for this trial in compliance with ICH GCP Guidelines, any regulatory requirements, and institutional requirements for the length of storage and for the protection of confidentiality of volunteers. The investigator will permit direct access to study records and source documents to authorized representatives of the sponsor, ethical committee(s) / institutional review board(s), regulatory agencies, authorised individuals from PATH, and the external monitor(s), for the purposes of quality assurance reviews, audits / inspections, and evaluation of the study safety and progress. Direct access includes examination, analysis, verification, and reproduction of de-identified records and reports that are important to the evaluation of the trial. Data and biological samples will be stored for 15 years.

11.1.2 Data collection

Designated trial staff will enter the data required by the protocol into the electronic CRF (eCRF).

11.1.3 Database management and quality control

All data should be recorded, handled and stored in a way that allows its accurate reporting, interpretation and verification.

An external monitor will review the data entered into the eCRFs by investigational staff for completeness and accuracy and will instruct the site personnel to make any required corrections or additions. Queries are made during each monitoring visit. Designated

investigator site staff are required to respond to the queries and confirm or correct the data. Medical history/current medical conditions and adverse events will be coded using the ICD-10 terminology.

11.2 Monitoring and Quality Assurance

Before study initiation, the protocol and eCRFs together with relevant SOPs will be reviewed by the sponsor, the investigators and their staff. During and after completion of the study, the data monitor will visit the site to check the completeness of records, the accuracy of entries on the eCRFs, the adherence to the protocol and to Good Clinical Practice, the progress of enrolment, and to ensure that sulfadoxine-pyrimethamine, piperaquine and Malarone® and are being dispensed and accounted for according to protocol.

The investigator will maintain source documents for each subject in the study, consisting of case and visit notes containing demographic and medical information, laboratory data, electrocardiograms, subject’s diaries, and the results of any other tests or assessments. All information on eCRFs must be traceable to these source documents in the subject's file. The only exception is the data from the quantitative PCR, which is loaded from the PCR machine directly into the eCRF. As with all parts of the eCRF, there is an audit trail in place to register every data entry. The investigator will also keep the original informed consent form signed by the subject (a signed copy is given to the subject).

The investigator will give the external monitor access to all relevant source documents to confirm their consistency with the eCRF entries. According to the NFU risk classification system, this clinical trial has been classified as ‘middle risk’. The monitor will perform full verification for the presence of informed consent, adherence to the inclusion/exclusion criteria and documentation of SAEs. The recording of data that will be used for all primary and safety variables will be assessed for 25% of included subjects (i.e. 8 subjects).

11.3 Amendments

Amendments are changes made to the research, and will only be made after favourable opinions / approvals by the CCMO and WIRB have been given - except where necessary to eliminate apparent immediate hazards to the subject(s). All amendments will be submitted to CCMO and WIRB for review and approval.

11.4 Annual progress report

The investigator will submit a summary of the progress of the trial to the CCMO once a year. Information will be provided on the date of inclusion of the first subject, numbers of subjects included and numbers of subjects that have completed the trial, serious adverse events/ serious adverse reactions, other problems, and amendments.

Continuing review reports will be submitted to WIRB, in accordance with its reporting requirements.

11.5 Temporary halt and (prematurely) end of study report

The investigator will notify the CCMO of the end of the study within a period of 8 weeks. The end of the study is defined as the subject's last visit on day 64 after the malaria challenge infection.

The investigator will notify the CCMO immediately of a temporary halt of the study, including the reason of such an action. In case the study is ended prematurely, the investigator will notify the CCMO within 15 days, including the reasons for the premature termination. Within one year after the end of the study, the investigator will submit a final study report with the results of the study, including any publications/abstracts of the study, to the CCMO. PATH REC and WIRB will also be notified of any decisions to prematurely suspend or terminate the study, and a study closure report will be submitted to WIRB when all subjects have finished their final visits and follow-up and when the sponsor has indicated the study is closed.

11.6 Public disclosure and publication policy

The final report will be prepared by the investigators at the Radboud university medical center. It will be signed by the project leader or the principal investigator. The investigators will make every effort to publish the results in a peer-reviewed journal.

12. STRUCTURED RISK ANALYSIS

12.1 Controlled Human Malaria Infection

12.1.1 Potential issues of concern

In this study all volunteers undergo a Controlled Human Malaria Infection by the bites of 5 laboratory reared *Anopheles* mosquitoes infected with the 3D7 clone of *Plasmodium falciparum*.

a. Level of knowledge about mechanism of action

The causative organisms of malaria, *Plasmodium* parasites, were first identified by Laveran in 1880 and their complete life-cycle in the mammalian host was elucidated by 1947. Extensive clinical experience of malaria infection has since been accumulated by the medical community. Nevertheless, certain aspects of the pathophysiology of (severe) malaria (e.g. cerebral malaria) remain incompletely understood. Such manifestations, however, do not occur during Controlled Human Malaria Infections due to the very early treatment of study subjects and hence the clearance of parasitaemia at extremely low levels (see also below).

b. Previous exposure of human beings with the test product(s) and/or products with a similar biological mechanism

There is extensive clinical experience with controlled human *P. falciparum* malaria infections by the bite of infected mosquitoes. Since 1986 more than 3,500 volunteers have had CHMI by the bites of mosquitoes fed on cultures of *P. falciparum* gametocytes to produce *P. falciparum* sporozoites. Worldwide the majority of these infections have been performed with the laboratory NF54 strain or its daughter clone 3D7. This has proved to be a reproducible, predictable and safe method of inducing *P. falciparum* infections. The results of such studies were summarized in 1997 [11], in 2007 [28] and in 2011 [76].

c. Can the primary or secondary mechanism be induced in animals and/or in *ex-vivo* human cell material?

In vitro assays cannot capture the complexity of the multi-stage life-cycle of *Plasmodium* parasites and their complex of interaction with the host. Although (parts of) the *Plasmodium* life-cycle can be reproduced in (rodent or primate) animal models, these either involve non-human *Plasmodium*-species or non-natural host-parasite combinations. As a result, extrapolation of pathophysiological and immunological aspects of infection to human disease are only possible to a limited extent [77, 78].

d. Selectivity of the mechanism to target tissue in animals and/or human beings

The life-cycle of malaria parasites follows a fixed and pre-determined course in the human host, including migration of sporozoites from the skin to the liver sinusoids, intra-hepatic development, asexual multiplication and finally gametocytogenesis within erythrocytes. These developments are constrained by multiple parasite-host ligand interactions and the parasite's ability to manipulate the host cell's internal environment [79].

e. Analysis of potential effect

Parasitaemia in subjects undergoing CHMIs will never exceed ±0.001% (i.e. equivalent to 50 parasites/µL, the detection level of thick smear microscopy is ~5 parasites/µL). In contrast, manifestations of severe malaria generally only occur above 5% parasitaemia and by definition never below 1% parasitaemia (WHO Guideline Severe Malaria, SWAB Richtlijn).

f. Pharmacokinetic considerations

Not applicable.

g. Study population

Included subjects are healthy young adult volunteers, who have been extensively screened for any evidence of co-morbidity, in particular cardiovascular risk factors. Female subjects of child-bearing age are screened for pregnancy by urine test and are required to use contraception throughout the study period.

h. Interaction with other products

Concurrent use of drugs potentially interacting with sulfadoxine-pyrimethamine (e.g trimpethoprim, trimethoprim-sulfonamide combinations, chloroquine, lorazepam, phenytoin, coumarin derivatives, folic acid antagonists), piperaquine (e.g. QTc-prolonging medication, strong CYP3A4 inhibitors, rifampicin, carbamazepine, phenytoin, phenobarbital, St.
John's wort (Hypericum perforatum)) and atovaquone-proguanil (e.g. artemether-lumefantrine, rifampicin, metoclopramide, oral anti-coagulants and certain anti-retrovirals) are contra-indicated.

No adverse drug interactions have been described between piperaquine, sulfadoxine-pyrimethamine and/or atovaquone-proguanil[80, 81][82, 83]. Piperaquine has been combined with sulfadoxine-pyrimethamine in clinical trials for intermittent preventive treatment and is safe, well tolerated, and effective [84][85].

The relatively long half-life of sulfadoxine-pyrimethamine and, especially, piperaquine might potentially cause a low concentration of either of these drugs at the time of final Malarone® treatment. However, no adverse interactions between these drugs have been described nor

are expected to occure in these doses[1, 82][81, 83]. Piperaquine is mainly metabolised by CYP3A4 and to a lesser extent by CYP2C9 and CYP2C19. Piperaquine is an inhibitor of CYP3A4 (also in a time-dependent manner) and to a lesser extent of CYP2C19, while it induced CYP2E1[83]. In Malarone®, there is no evidence that atovaquone (the primary effective anti-malarial component) is metabolised and there is negligible excretion of atovaquone in urine with the parent drug being predominantly (> 90%) eliminated unchanged in faeces. Proguanil hydrochloride is partially metabolised with less than 40% being excreted unchanged in the urine. Proguanil is metabolized to cycloguanil (primarily via CYP2C19) and 4-chlorophenylbiguanide, and these are also excreted unchanged in the urine. Proguanil also has antimalarial activity independent of its metabolism to cycloguanil, and proguanil, but not cycloguanil, is able to potentiate the ability of atovaquone to collapse mitochondrial membrane potential in malaria parasites. During administration of Malarone® at recommended doses, proguanil metabolism status appears to have no implications for treatment or prophylaxis of malaria[81].

Therefore, it is highly unlikely that low concentration piperaquine would affect the effectivity or safety of Malarone® in treatment of malaria.

i. Predictability of effect

In this trial all volunteers will experience blood stage malaria infection. Blood stage malaria infection has been seen after CHMI in over 3,500 volunteers. The progression and symptoms of this parasitaemia has been demonstrated to be reproducible and predictable [11, 28, 29]. However, the occurrence of cardiac serious adverse events in Radboudumc CHMI trials has led to an increase in safety measures, discussed in a separate section below.

j. Can effects be managed?

Subjects are followed up intensively on an outpatient basis for clinical and parasitological assessment to ensure treatment is started at the earliest possible time point. The 3D7 *P. falciparum* clone has been tested for sensitivity to sulfadoxine-pyrimethamine, piperaquine and atovaquone-proguanil *in vitro*. Should treatment with this drug need to be discontinued prematurely in any subject for whatever other reason (e.g. intolerability), various other anti-malarial drugs are available, both oral and intravenous, to which this clone is also susceptible.

12.1.2 Cardiac events following Controlled Human Malaria Infections

In past Radboudumc CHMIs, three cardiac events have occurred after infection with the NF54 strain with the confirmed or differential diagnosis of myocarditis.

Case 1

In 2007 a 20 year old female was immunized with the candidate malaria vaccine, LSA3 (NL14715.000.06), underwent a CHMI and was treated with artemether/lumefantrine (Riamet®) after a positive thick smear. She presented with retrosternal chest pain three days after treatment, blood slide was negative. Elevated troponin-I (maximally 11.80ug/l) and ECG abnormalities were suggestive for a cardiac event with minimal cardiac damage and an MRI showed no abnormalities. Risk factors for cardiac disease in this volunteer included an acute myocardial infarction in a paternal grandfather at age 43 and a paternal family history of dyslipidemia. The final diagnosis was acute coronary syndrome or myocarditis. This case is reported in [86], attachment K4a. Following this event, recommendations of the European Malaria Vaccine Development Association and the CCMO followed for improved safety of participating volunteers:

- Riamet® is no longer used as treatment during CHMIs at Radboudumc. Malarone® replaces it.
- Individuals will be excluded from participation if they have 1st or 2nd degree relatives who had cardiac events when less than 50 years of age.
- Volunteers will be required to stay very close to the Radboudumc to ensure maximal safety from day 5 after CHMI until treatment has been finished (maximum 12 days).
- Monitoring of highly sensitive troponin T (hsTropT), D-dimer, lactate dehydrogenase (LDH) and thrombocytes.

Case 2

In 2013, a healthy male, taking part in the TIP5 clinical trial (NL39541.091.12) underwent a CHMI challenge after immunization with cyropreserved sporozoites under chloroquine prophylaxis (CPS protocol). Thick smear became positive after challenge infection and the volunteer was treated with atovaquone/proguanil (Malarone®). Per protocol blood examination revealed an elevated troponin-T (maximally 1115ng/l) on the second day of Malarone® treatment, while the volunteer was asymptomatic. He was admitted to cardiology and troponin-T values, ECG abnormalities and a cardiac MRI confirmed the diagnosis acute myocarditis. During admission he experienced a 20 minute episode of retrosternal chest pain. Further diagnostic tests and patient history revealed a concurrent rhinovirus infection and a boosting regime of standard travel vaccines 46 days preceding the CHMI, with a booster 20 days before the CHMI. This case has been reported in [87], attachment K4b. Due to this SAE, safety recommendations were integrated into our procedures for CHMI. These included:

- Exclusion of volunteers who took standard vaccinations within 3 months before the start of the trial or are planning to take standard vaccinations during the trial period up to 8 weeks after CHMI.
- Increased control of hs troponin T as a marker of cardiac damage; initiation of treatment with Malarone® when hs troponin T > 0.1 µg/ml or on recommendation of the cardiologist.
- Continuation of daily LDH measurements starting from day 5 after CHMI; Malarone® treatment will be initiated if LDH values are above 1000 U/L.
- Daily measurements of thrombocytes; volunteers will be treated with Malarone® when thrombocyte levels are < 120x 10^9/L.
- After CHMI we will rely on real-time qPCR for diagnosis of malaria and initiation of treatment, using the following criteria:
  - Two consecutive positive qPCR results in a volunteer with temperature <38.0^{0}C
  - One positive qPCR result in a volunteer with temperature ≥38.0^{0}C
  - One positive thick smear (which will be made if a volunteers attends for evening follow-up with temperature ≥38.0^{0}C or on decision of the trial clinician)

Case 3

In 2014, a healthy, 23 year old, male volunteer underwent a CHMI under chloroquine prophylaxis (CPS-immunization). On day 10 after exposure to bites of 15 malaria infected mosquitoes, routine per protocol blood examinations revealed an elevated troponin-T (maximally 168ng/l) though the volunteer was asymptomatic. This volunteer had no obvious risk factors for cardiac disease other than cigarette smoking and recent cannabis use. This volunteer was negative by thick smear but positive by PCR with maximum parasitaemia was

1265Pf/ml while under chloroquine prophylaxis. Following this cardiac event, further safety measures have been integrated into our CHMI protocols:

- Volunteers will be treated with antimalarial after a single positive qPCR after malaria challenge infection.
- Use of cannabis will be added as an exclusion criterion
- Excessive physical exercise around immunization and during challenge period will be prohibited.

12.1.3 Synthesis

There is a large clinical experience with infecting humans by the bite of *P. falciparum*-infected mosquitoes. Since 1986 more than 3,500 volunteers have had CHMI by the bites of mosquitoes fed on cultures of *P. falciparum* gametocytes to produce sporozoites [27]. This has proved to be a reproducible, predictable and safe method of inducing *P. falciparum* malaria. The results of such studies were summarized in 1997 [11], in 2007 [28] and in 2011 [29].

Following three cardiac events after blood stage parasitaemia, our safety procedures for CHMI have been strongly intensified. In the current trial, we will use a 3D7 clone and adhere to those stringent procedures that are relevant, see section 1.4.

13. REFERENCES

1. World Health, O., *World Malaria Report*. 2014.
2. Ashley, E.A., et al., *Spread of artemisinin resistance in Plasmodium falciparum malaria.* N Engl J Med, 2014. **371**(5): p. 411-23.
3. Sauerwein, R.W. and T. Bousema, *Transmission blocking malaria vaccines: Assays and candidates in clinical development.* Vaccine, 2015.
4. Mal, E.R.A.C.G.o.B.S., *Consultative Group on Vaccines. A research agenda for malaria eradication: vaccines.* PLoS Med, 2011. **8**(1): p. e1000398.
5. Adjalley, S.H., et al., *Quantitative assessment of Plasmodium falciparum sexual development reveals potent transmission-blocking activity by methylene blue.* Proc Natl Acad Sci U S A, 2011. **108**(47): p. E1214-23.
6. Bhatt, S., et al., *The effect of malaria control on Plasmodium falciparum in Africa between 2000 and 2015.* Nature, 2015. **526**(7572): p. 207-11.
7. Hoffman, S.L., et al., *The March Toward Malaria Vaccines.* Am J Prev Med, 2015. **49**(6S4): p. S319-S333.
8. Griffin, J.T., et al., *Reducing Plasmodium falciparum malaria transmission in Africa: a model-based evaluation of intervention strategies.* PLoS Med, 2010. **7**(8).
9. Targett, G.A. and B.M. Greenwood, *Malaria vaccines and their potential role in the elimination of malaria.* Malar J, 2008. **7 Suppl 1**: p. S10.
10. Kiszewski, A.E., *Blocking Plasmodium falciparum Malaria Transmission with Drugs: The Gametocytocidal and Sporontocidal Properties of Current and Prospective Antimalarials.* Pharmaceuticals, 2010. **4**(1): p. 44-68.
11. Church, L.W., et al., *Clinical manifestations of Plasmodium falciparum malaria experimentally induced by mosquito challenge.* J Infect Dis, 1997. **175**(4): p. 915-20.
12. Epstein, J.E., et al., *Safety and clinical outcome of experimental challenge of human volunteers with Plasmodium falciparum-infected mosquitoes: an update.* J Infect Dis, 2007. **196**(1): p. 145-54.
13. Roestenberg, M., et al., *Long-term protection against malaria after experimental sporozoite inoculation: an open-label follow-up study.* Lancet, 2011. **377**(9779): p. 1770-6.
14. Sauerwein, R.W., M. Roestenberg, and V.S. Moorthy, *Experimental human challenge infections can accelerate clinical malaria vaccine development.* Nat Rev Immunol, 2011. **11**(1): p. 57-64.
15. Alving, A.S., et al., *Potentiation of the curative action of primaquine in vivax malaria by quinine and chloroquine.* J Lab Clin Med, 1955. **46**(2): p. 301-6.
16. Shapiro, T.A., et al., *Prophylactic activity of atovaquone against Plasmodium falciparum in humans.* Am J Trop Med Hyg, 1999. **60**(5): p. 831-6.
17. Berman, J.D., et al., *Causal prophylactic efficacy of atovaquone-proguanil (Malarone) in a human challenge model.* Trans R Soc Trop Med Hyg, 2001. **95**(4): p. 429-32.
18. Beadle, C., et al., *Diagnosis of malaria by detection of Plasmodium falciparum HRP-2 antigen with a rapid dipstick antigen-capture assay.* Lancet, 1994. **343**(8897): p. 564-8.
19. McCall, M.B., et al., *Plasmodium falciparum infection causes proinflammatory priming of human TLR responses.* J Immunol, 2007. **179**(1): p. 162-71.
20. Teirlinck, A.C., et al., *Longevity and composition of cellular immune responses following experimental Plasmodium falciparum malaria infection in humans.* PLoS Pathog, 2011. **7**(12): p. e1002389.
21. Bousema, T. and C. Drakeley, *Epidemiology and infectivity of Plasmodium falciparum and Plasmodium vivax gametocytes in relation to malaria control and elimination.* Clin Microbiol Rev, 2011. **24**(2): p. 377-410.
22. Butcher, G.A., *Antimalarial drugs and the mosquito transmission of Plasmodium.* Int J Parasitol, 1997. **27**(9): p. 975-87.
23. Sawa, P., et al., *Malaria transmission after artemether-lumefantrine and dihydroartemisinin-piperaquine: a randomized trial.* J Infect Dis, 2013. **207**(11): p. 1637-45.

24. Bousema, T., et al., *Can field-based mosquito feeding assays be used for evaluating transmission-blocking interventions?* Trends Parasitol, 2013. **29**(2): p. 53-9.
25. McCarthy, J., *Investigating the Controlled Human Malaria Infection model as a platform to evaluate transmission blocking interventions*. 2014: American Society of Tropical Medicine and Hygiene Annual Meeting, New Orleans.
26. Laurens, M.B., et al., *A consultation on the optimization of controlled human malaria infection by mosquito bite for evaluation of candidate malaria vaccines.* Vaccine, 2012. **30**(36): p. 5302-4.
27. Chulay, J.D., et al., *Malaria transmitted to humans by mosquitoes infected from cultured Plasmodium falciparum.* Am J Trop Med Hyg, 1986. **35**(1): p. 66-8.
28. Epstein, J.E., et al., *Malaria vaccines: are we getting closer?* Curr Opin Mol Ther, 2007. **9**(1): p. 12-24.
29. Roestenberg, M., et al., *Comparison of clinical and parasitological data from controlled human malaria infection trials.* PLoS One, 2012. **7**(6): p. e38434.
30. Hermsen, C.C., et al., *Testing vaccines in human experimental malaria: statistical analysis of parasitemia measured by a quantitative real-time polymerase chain reaction.* Am J Trop Med Hyg, 2004. **71**(2): p. 196-201.
31. Schneider, P., et al., *Quantification of Plasmodium falciparum gametocytes in differential stages of development by quantitative nucleic acid sequence-based amplification.* Mol Biochem Parasitol, 2004. **137**(1): p. 35-41.
32. Schneider, P., et al., *Quantification of female and male Plasmodium falciparum gametocytes by reverse transcriptase quantitative PCR.* Mol Biochem Parasitol, 2015. **199**(1-2): p. 29-33.
33. Mulder, B., et al., *Plasmodium falciparum: membrane feeding assays and competition ELISAs for the measurement of transmission reduction in sera from Cameroon.* Exp Parasitol, 1999. **92**(1): p. 81-6.
34. Schneider, P., et al., *(Sub)microscopic Plasmodium falciparum gametocytaemia in Kenyan children after treatment with sulphadoxine-pyrimethamine monotherapy or in combination with artesunate.* Int J Parasitol, 2006. **36**(4): p. 403-8.
35. Marquart, L., et al., *Evaluating the pharmacodynamic effect of antimalarial drugs in clinical trials by quantitative PCR.* Antimicrob Agents Chemother, 2015. **59**(7): p. 4249-59.
36. Falade, C.O., et al., *Intermittent preventive treatment with sulphadoxine-pyrimethamine is effective in preventing maternal and placental malaria in Ibadan, south-western Nigeria.* Malar J, 2007. **6**: p. 88.
37. Peters, P.J., et al., *Safety and toxicity of sulfadoxine/pyrimethamine: implications for malaria prevention in pregnancy using intermittent preventive treatment.* Drug Saf, 2007. **30**(6): p. 481-501.
38. van Eijk, A.M., et al., *Effectiveness of intermittent preventive treatment with sulphadoxine-pyrimethamine for control of malaria in pregnancy in western Kenya: a hospital-based study.* Trop Med Int Health, 2004. **9**(3): p. 351-60.
39. Zongo, I., et al., *Randomized Noninferiority Trial of Dihydroartemisinin-Piperaquine Compared with Sulfadoxine-Pyrimethamine plus Amodiaquine for Seasonal Malaria Chemoprevention in Burkina Faso.* Antimicrob Agents Chemother, 2015. **59**(8): p. 4387-96.
40. Roche, *Fansidar product information*. 2005.
41. Hogh, B., et al., *The differing impact of chloroquine and pyrimethamine/sulfadoxine upon the infectivity of malaria species to the mosquito vector.* Am J Trop Med Hyg, 1998. **58**(2): p. 176-82.
42. Sowunmi, A., et al., *Effects of antifolates--co-trimoxazole and pyrimethamine-sulfadoxine--on gametocytes in children with acute, symptomatic, uncomplicated, Plasmodium falciparum malaria.* Mem Inst Oswaldo Cruz, 2005. **100**(4): p. 451-5.
43. Gargano, N., F. Cenci, and Q. Bassat, *Antimalarial efficacy of piperaquine-based antimalarial combination therapies: facts and uncertainties.* Trop Med Int Health, 2011. **16**(12): p. 1466-73.
44. Davis, T.M., et al., *Piperaquine: a resurgent antimalarial drug.* Drugs, 2005. **65**(1): p. 75-87.

45. Nosten, F. and N.J. White, *Artemisinin-based combination treatment of falciparum malaria.* Am J Trop Med Hyg, 2007. **77**(6 Suppl): p. 181-92.
46. Karunajeewa, H.A., et al., *Pharmacokinetics and efficacy of piperaquine and chloroquine in Melanesian children with uncomplicated malaria.* Antimicrob Agents Chemother, 2008. **52**(1): p. 237-43.
47. Myint, H.Y., et al., *Efficacy and safety of dihydroartemisinin-piperaquine.* Trans R Soc Trop Med Hyg, 2007. **101**(9): p. 858-66.
48. Group, W.A.R.N.W.D.S., *The effect of dosing regimens on the antimalarial efficacy of dihydroartemisinin-piperaquine: a pooled analysis of individual patient data.* PLoS Med, 2013. **10**(12).
49. Sim, I.K., T.M. Davis, and K.F. Ilett, *Effects of a high-fat meal on the relative oral bioavailability of piperaquine.* Antimicrob Agents Chemother, 2005. **49**(6): p. 2407-11.
50. Nguyen, T.C., et al., *Pharmacokinetics of the antimalarial drug piperaquine in healthy Vietnamese subjects.* Am J Trop Med Hyg, 2008. **79**(4): p. 620-3.
51. Price, R.N., G. Dorsey, and F. Nosten, *Antimalarial therapies in children from Papua New Guinea.* N Engl J Med, 2009. **360**(12): p. 1254; author reply 1255.
52. Hai, T.N., et al., *The influence of food on the pharmacokinetics of piperaquine in healthy Vietnamese volunteers.* Acta Trop, 2008. **107**(2): p. 145-9.
53. Yeka, A., et al., *Artemether-lumefantrine versus dihydroartemisinin-piperaquine for treating uncomplicated malaria: a randomized trial to guide policy in Uganda.* PLoS One, 2008. **3**(6): p. e2390.
54. Kamya, M.R., et al., *Artemether-lumefantrine versus dihydroartemisinin-piperaquine for treatment of malaria: a randomized trial.* PLoS Clin Trials, 2007. **2**(5): p. e20.
55. Nambozi, M., et al., *Safety and efficacy of dihydroartemisinin-piperaquine versus artemether-lumefantrine in the treatment of uncomplicated Plasmodium falciparum malaria in Zambian children.* Malar J, 2011. **10**: p. 50.
56. Ratcliff, A., et al., *Two fixed-dose artemisinin combinations for drug-resistant falciparum and vivax malaria in Papua, Indonesia: an open-label randomised comparison.* Lancet, 2007. **369**(9563): p. 757-65.
57. Karunajeewa, H., et al., *Safety evaluation of fixed combination piperaquine plus dihydroartemisinin (Artekin) in Cambodian children and adults with malaria.* Br J Clin Pharmacol, 2004. **57**(1): p. 93-9.
58. Luntamo, M., et al., *Effect of repeated treatment of pregnant women with sulfadoxine-pyrimethamine and azithromycin on preterm delivery in Malawi: a randomized controlled trial.* Am J Trop Med Hyg, 2010. **83**(6): p. 1212-20.
59. Maokola, W., et al., *Safety of sulfadoxine/pyrimethamine for intermittent preventive treatment of malaria in infants: evidence from large-scale operational research in southern Tanzania.* Int Health, 2011. **3**(3): p. 154-9.
60. Mytton, O.T., et al., *Electrocardiographic safety evaluation of dihydroartemisinin piperaquine in the treatment of uncomplicated falciparum malaria.* Am J Trop Med Hyg, 2007. **77**(3): p. 447-50.
61. EMA, *Assessment Report Eurartesim*. 2011.
62. EMA, *Annex 1 Summary of Product Characteristics: Product Information for Eurartesim*. 2011.
63. Targett, G., et al., *Artesunate reduces but does not prevent posttreatment transmission of Plasmodium falciparum to Anopheles gambiae.* J Infect Dis, 2001. **183**(8): p. 1254-9.
64. Hallett, R.L., et al., *Chloroquine/sulphadoxine-pyrimethamine for gambian children with malaria: transmission to mosquitoes of multidrug-resistant Plasmodium falciparum.* PLoS Clin Trials, 2006. **1**(3): p. e15.
65. Ouédraogo. *A protocol for membrane feeding assays to determine the infectiousness of P. falciparum naturally infected individuals to Anopheles gambiae*
2013; Available from: http://www.malariaworld.org/sites/default/files/mwjournal/article/MWJ%202013_4_16.pdf.
66. Blagborough, A.M., et al., *Transmission-blocking interventions eliminate malaria from laboratory populations.* Nat Commun, 2013. **4**: p. 1812.
67. Churcher, T.S., et al., *Measuring the blockade of malaria transmission--an analysis of the Standard Membrane Feeding Assay.* Int J Parasitol, 2012. **42**(11): p. 1037-44.

68. Stone, W.J., et al., *The relevance and applicability of oocyst prevalence as a read-out for mosquito feeding assays.* Sci Rep, 2013. **3**: p. 3418.
69. Stone, W., et al., *A comparison of Plasmodium falciparum circumsporozoite protein-based slot blot and ELISA immuno-assays for oocyst detection in mosquito homogenates.* Malar J, 2015. **14**(1): p. 451.
70. Wampfler, R., et al., *Strategies for detection of Plasmodium species gametocytes.* PLoS One, 2013. **8**(9): p. e76316.
71. Tiburcio, M., et al., *Specific expression and export of the Plasmodium falciparum Gametocyte EXported Protein-5 marks the gametocyte ring stage.* Malar J, 2015. **14**: p. 334.
72. Joice, R., et al., *Inferring developmental stage composition from gene expression in human malaria.* PLoS Comput Biol, 2013. **9**(12): p. e1003392.
73. Karl, S., et al., *Enhanced detection of gametocytes by magnetic deposition microscopy predicts higher potential for Plasmodium falciparum transmission.* Malar J, 2008. **7**: p. 66.
74. Karl, S., T.M. Davis, and T.G. St-Pierre, *A comparison of the sensitivities of detection of Plasmodium falciparum gametocytes by magnetic fractionation, thick blood film microscopy, and RT-PCR.* Malar J, 2009. **8**: p. 98.
75. Jones, S., et al., *Naturally acquired antibody responses to recombinant Pfs230 and Pfs48/45 transmission blocking vaccine candidates.* J Infect, 2015. **71**(1): p. 117-27.
76. Roestenberg, M., et al., *Efficacy of preerythrocytic and blood-stage malaria vaccines can be assessed in small sporozoite challenge trials in human volunteers.* J Infect Dis, 2012. **206**(3): p. 319-23.
77. Craig, A.G., et al., *The role of animal models for research on severe malaria.* PLoS Pathog, 2012. **8**(2): p. e1002401.
78. Langhorne, J., et al., *The relevance of non-human primate and rodent malaria models for humans.* Malar J, 2011. **10**: p. 23.
79. Gaur, D. and C.E. Chitnis, *Molecular interactions and signaling mechanisms during erythrocyte invasion by malaria parasites.* Curr Opin Microbiol, 2011. **14**(4): p. 422-8.
80. Organization, W.H., *Guidelines for the treatment of malaria. Third edition*. 2015.
81. UK, G., *Summary of Product Characteristics Malarone*. 2013: Middlesex, UK. p. 8.
82. Pharma, R., *Drug prescription information Fansidar®*. 2006: Reinach, Switserland.
83. (MMV), M.f.M.V., *INVESTIGATOR'S SUMMARY - Piperaquine phosphate*. 2012.
84. Benjamin, J.M., et al., *Population pharmacokinetics, tolerability, and safety of dihydroartemisinin-piperaquine and sulfadoxine-pyrimethamine-piperaquine in pregnant and nonpregnant Papua New Guinean women.* Antimicrob Agents Chemother, 2015. **59**(7): p. 4260-71.
85. Cisse, B., et al., *Randomized trial of piperaquine with sulfadoxine-pyrimethamine or dihydroartemisinin for malaria intermittent preventive treatment in children.* PLoS One, 2009. **4**(9): p. e7164.
86. Nieman, A.E., et al., *Cardiac complication after experimental human malaria infection: a case report.* Malar J, 2009. **8**: p. 277.
87. van Meer, M.P., et al., *Idiopathic acute myocarditis during treatment for controlled human malaria infection: a case report.* Malar J, 2014. **13**: p. 38.
